# Integrative bioinformatics informed by network toxicology and machine learning elucidates the carcinogenic mechanisms of benzo[a]pyrene-induced breast cancer

**DOI:** 10.7717/peerj.21346

**Published:** 2026-06-16

**Authors:** Run Qu, Jing Zou, Qingfen Ruan, Ruiqin Han, Canmei Li, Yi Liang, Yanhong Zhao, Yuzhe Zhang

**Affiliations:** 1 School of Basic Medical Sciences, Dali University, Dali, China; 2 The First Affiliated Hospital, Department of Respiratory Medicine, Dali University, Dali, China; 3 The First Affiliated Hospital, Department of Gastroenterology, Dali University, Dali, China; 4 Institute of Basic Medical Sciences, Chinese Academy of Medical Sciences & Peking Union Medical College, Beijing, China; 5 Department of Oncology, Dali Bai Autonomous Prefecture People’s Hospital, Dali, China; 6 Princess Margaret Cancer Centre, University Health Network TMDT-MaRS Centre, Toronto, Canada; 7 Yunnan Key Laboratory of Screening and Research on Anti-pathogenic Plant Resources from Western Yunnan, Dali, China

**Keywords:** Benzo[a]pyrene, Network toxicology, Molecular docking, Immune infiltration profiling, Breast cancer

## Abstract

**Background:**

Benzo[a]pyrene (BaP) is a recognized mutagen and carcinogen, yet epidemiological links to breast cancer (BC) remain inconclusive.

**Methods:**

We integrated network toxicology, machine learning, and bioinformatics. BaP targets (ChEMBL, PharmMapper, SEA, GeneCards, OMIM) were intersected with BC genes, followed by Gene Ontology (GO) and Kyoto Encyclopedia of Genes and Genomes (KEGG) enrichment. Core regulatory genes were further screened using diverse machine-learning algorithms, and their expression levels, diagnostic performance, and associations with the tumor immune microenvironment were subsequently validated. Gene Set Variation Analysis (GSVA) was applied to assess pathway activity, and Cytoscape was used to construct a lncRNA–miRNA–mRNA multilevel regulatory network, thereby elucidating post-transcriptional control mechanisms. Finally, molecular docking and molecular dynamics simulations were performed to evaluate potential interactions between BaP and the core targets.

**Results:**

A total of 216 overlapping BaP–breast cancer targets were initially identified, which were significantly enriched in processes such as the mitogen-activated protein kinase (MAPK) signaling pathway. Seven core genes were identified by machine-learning–based screening; among them, KIF11, INHBA, NEK2, and AURKA exhibited significantly higher expression in breast cancer tissues and were associated with worse patient prognosis and altered immune-cell infiltration. Based on pathway analyses, tumor progression was inferred to be promoted by these genes through regulation of the cell cycle, DNA replication, and cell-adhesion pathways. Molecular modeling indicated that BaP could form stable binding conformations with the proteins encoded by KIF11, AURKA, INHBA, and NEK2, suggesting possible direct interactions.

**Conclusion:**

This research provides new theoretical insights into the etiology of environmental pollutant-induced BC and offers potential molecular biomarkers for risk assessment and the formulation of targeted prevention strategies.

## Introduction

Breast cancer (BC) is the most prevalent malignancy and a leading cause of cancer-related mortality in women. In 2020, approximately 2.3 million new cases were reported, underscoring its global health burden (Sung et al., 2021; Cai et al., 2025). While genetic predisposition, hormonal influences, and lifestyle factors have been established as major contributors, these traditional risk factors do not fully explain the persistently high incidence of BC. Increasing evidence highlights the significant role of environmental exposures in carcinogenesis. With estimates suggesting that environmental factors account for 70%–90% of cancer risk, compared with 10%–30% attributable to genetic factors (Wu et al., 2016). Environmental pollutants have emerged as critical drivers of BC initiation and progression through mechanisms such as DNA damage and epigenetic dysregulation (Hill & Hodsdon, 2014; Genchi et al., 2020).

Outdoor air pollution has been classified as carcinogenic to humans (Group 1) by the International Agency for Research on Cancer (IARC) (Gray et al., 2017; White, Bradshaw & Hamra, 2018). Among its major constituents, polycyclic aromatic hydrocarbons (PAHs)—particularly benzo[a]pyrene (BaP)—exhibit carcinogenic, mutagenic, and endocrine-disrupting activities (Amadou et al., 2021). BaP is a five-ring PAH with a highly stable chemical structure. The high bond energies of its carbon–carbon bonds and its low bioavailability limit microbial metabolism and hinder environmental degradation. As a result, BaP persists in ecosystems and is continually introduced through incomplete combustion, including industrial emissions, vehicle exhaust, and tobacco smoke (Adeniran et al., 2021; Barangi et al., 2023).

With respect to toxicological mechanisms, BaP-induced cytotoxicity, genotoxicity, neurotoxicity, mutagenicity, and carcinogenicity have been documented across multiple tissues and cell types (Myers et al., 2021; Bukowska, Mokra & Michałowicz, 2022; Yi et al., 2025). Specifically, Benzo[a]pyrene-7,8-diol-9,10-epoxide (BPDE), a highly reactive ultimate carcinogen, arises when BaP is metabolized by cytochrome P450 enzymes, primarily CYP1A1 and CYP1B1. BPDE forms covalent adducts with DNA, leading to mutations in tumor-suppressor genes such as TP53. This molecular mechanism provides crucial evidence for its carcinogenicity (Malik, David & Gooderham, 2018; Stading et al., 2021). Furthermore, a significant dose–response association between environmental air pollutant exposure and health outcomes has been evidenced in epidemiologic research, including tobacco smoke and BC incidence (Mordukhovich et al., 2016; White, Bradshaw & Hamra, 2018). Beyond DNA damage, oncogenic signaling pathways are differentially modulated by BaP—for example, mitogen-activated protein kinase (MAPK) activity (ERK/JNK/p38) and regulation of the cyclin D1/CDK4 complex—*via* an AhR-mediated Src/ERK axis, thereby promoting aberrant cell proliferation (Wang et al., 2020; Latchford et al., 2025). Nevertheless, the molecular mechanisms linking BaP to BC remain insufficiently elucidated, particularly due to a paucity of systematic studies identifying its key targets and the interaction networks of associated pathways.

Accordingly, we hypothesized that BaP exposure may contribute to BC pathogenesis by interfering with the expression of genes involved in critical biological processes such as cell cycle regulation and immune response. To systematically investigate this hypothesis, we employed an integrative in silico strategy combining network toxicology, machine learning-based prioritization, molecular docking, and molecular dynamics (MD) simulations to evaluate potential molecular associations between BaP-related targets and BC. With continued advances in toxicology, such integrative computational approaches have been increasingly adopted to systematically identify toxicological targets and regulatory pathways (Hartung et al., 2017; Huang, 2024). In parallel, integration of multi-omics data with machine-learning models has improved the accuracy of toxicity prediction (Jung, Kim & Cho, 2022), facilitated the discovery of novel molecular targets, and offered new perspectives on environmentally induced diseases. This approach is increasingly regarded as a new research paradigm for health risk assessment.

In this study, we first identified the shared targets between BaP and BC. We then screened key candidate targets and pathways that may play a role in the molecular mechanisms linking BaP exposure to BC. Similar strategies have been successfully applied to investigate the carcinogenic mechanisms of other environmental contaminants in BC, such as acrylamide and di(2-ethylhexyl) phthalate (DEHP), highlighting the robustness and broad applicability of these methodologies (Duan et al., 2025; Wang & Wang, 2025). Furthermore, network toxicology has been utilized to explore putative molecular links between environmental BaP exposure and various diseases, including periodontitis and chronic obstructive pulmonary disease (COPD), where analyses identified core targets and validated interactions through molecular docking (Deng et al., 2025; Wenjie et al., 2025).

Building on these methodologies, the present study extends their application to investigate the associations between BaP exposure and BC. Through comprehensive multidimensional bioinformatics analyses, we systematically investigated the underlying molecular mechanisms linking BaP exposure to BC pathogenesis. Given the central role of immune dysregulation in tumor progression, we also performed immune infiltration analyses to examine whether BaP-associated molecular features correlate with variations in the tumor immune microenvironment—a dimension less attended in prior contaminant-focused network studies. Finally, molecular docking and MD simulations were conducted to characterize the predicted binding modes and structural stability of complexes between BaP and prioritized protein targets, thereby evaluating the plausibility of direct molecular interactions. Collectively, while previous works have established the utility of network toxicology for environmental toxicant-disease associations, our findings provide specific mechanistic insights into how environmental BaP exposure may contribute to BC pathogenesis through targets, immune-related perturbations, and stable direct binding, offering a stronger rationale for subsequent experimental validation and mechanistic clarification.

## Materials and Methods

### Screening and acquisition of potential BaP targets

Our study retrieved “Benzo[a]pyrene” from the PubChem database (https://pubchem.ncbi.nlm.nih.gov/) to obtain its chemical structure, SMILES identifier, and other fundamental information. Based on these search results, potential targets were predicted from the ChEMBL, PharmMapper, and Similarity Ensemble Approach (SEA) databases, specifying Homo sapiens as the reference species (He et al., 2024; Yadav et al., 2025). The predicted targets were standardized and corrected for gene nomenclature using the UniProt database (https://www.uniprot.org/). Finally, target data from all sources were integrated, redundancies were eliminated, and a comprehensive library of potential BaP targets was established.

### Screening of BC-Related targets

Potential targets associated with breast cancer (BC) were systematically collected by retrieving published literature and searching the GeneCards (https://www.genecards.org/) and OMIM (https://www.omim.org/) databases using “breast cancer” as the keyword. To ensure high disease relevance, the score threshold was set to the median value, and retained only genes exceeding this value to construct the BC–related target set. The Venny online tool was then used to generate a Venn diagram, and intersection genes between BaP and BC targets were extracted as key regulatory targets for subsequent analysis.

### Identification of DEGs

Differentially expressed genes (DEGs) between breast cancer tissues and adjacent normal tissues were identified using R software (version 4.5.1; R Core Team, 2025). Based on the limma package, an empirical Bayes method was applied to screen significant DEGs. Genes with an adjusted *P* value < 0.05 and an absolute log2 fold change (—log2FC—) > 0.585 were considered statistically significant.

### Gene Ontology and Kyoto Encyclopedia of Genes and Genomes enrichment analysis

To elucidate the biological basis of the association between BaP exposure and BC, potential targets were examined through Gene Ontology (GO) and Kyoto Encyclopedia of Genes and Genomes (KEGG) pathway enrichment analyses using the DAVID platform. GO analyses encompassed three categories—biological process (BP), cellular component (CC), and molecular function (MF)—to comprehensively characterize the biological attributes of these targets. KEGG pathway analysis was employed to identify key signaling pathways associated with the targets (Muley, 2025). Finally, we prioritized categories by *P* value and ultimately selected ten GO items and twenty KEGG pathways to visualize the enrichment.

### Core target screening based on machine learning algorithms

For the identification of biomarkers relevant to BC diagnosis, two machine-learning approaches were applied to feature-gene screening. First, candidate genes were initially screened using the Least Absolute Shrinkage and Selection Operator (LASSO) regression algorithm implemented in the “glmnet” package in R, in order to reduce dimensionality and prevent overfitting (Sun et al., 2021). Subsequently, the Support Vector Machine–Recursive Feature Elimination (SVM-RFE) algorithm was applied using the “e1071” package to further refine the feature set and enhance the model’s generalization ability (Huang et al., 2014).

Notably, LASSO (a linear, penalty-based method) and SVM-RFE (a margin-based wrapper approach) capture complementary aspects of feature importance (Li et al., 2023); therefore, taking the intersection of their outputs can mitigate method-specific bias and yield more stable candidate genes. Although ensemble- or tree-based methods (*e.g.*, random forest or gradient boosting) are also widely used for feature selection, their importance estimates may be less readily interpretable and can be sensitive to hyperparameter settings, particularly in settings with limited sample sizes and highly correlated predictors. Given our emphasis on interpretability and robustness, we therefore prioritized these two well-established, complementary methods and defined the overlapping genes as high-confidence core targets using the “VennDiagram” package for subsequent analyses.

### Differential expression and diagnostic validation of core gene

Gene expression profiles of 123 breast cancer tissues and 36 adjacent normal tissues were downloaded from the GEO database (GSE86374), which is a microarray-based dataset. Based on the GEO sample metadata for GSE86374, adjacent normal tissues were available for a subset of patients, and tumor–adjacent normal pairing could be identified for those cases; therefore, the dataset is partially paired rather than fully paired. To visualize the overall expression distributions, box plots were generated for candidate genes in breast cancer *versus* adjacent normal tissues. For unpaired tumor *vs* adjacent normal samples, the Wilcoxon rank-sum test was used (*P* < 0.05), while paired cases were analyzed with the Wilcoxon signed-rank test. Subsequently, receiver operating characteristic (ROC) curves were constructed for these differentially expressed genes, and the area under the curve (AUC) value was calculated to assess the diagnostic performance of each gene in distinguishing breast cancer from adjacent normal tissues.

### External dataset validation

To further validate the expression patterns and diagnostic potential of the four core genes (AURKA, NEK2, KIF11, and INHBA) in BC, we downloaded RNA-seq-based transcriptome profiling data and corresponding clinical information from the The Cancer Genome Atlas Breast Invasive Carcinoma (TCGA-BRCA) cohort in the The Cancer Genome Atlas (TCGA) Genomic Data Commons (GDC) portal (https://portal.gdc.cancer.gov/). This dataset includes 1118 breast cancer tissue samples and 113 adjacent normal tissue samples. The expression levels of the four core genes were extracted using the R toolkit and visualized with box plots. Receiver operating characteristic (ROC) curves were generated, and area under the curve (AUC) values were calculated to evaluate their diagnostic efficacy. The Cancer Genome Atlas Breast Invasive Carcinoma (TCGA-BRCA) cohort provided independent external validation, complementing the internal validation performed with the Gene Expression Omnibus (GEO) dataset.

### lncRNA-miRNA-mRNA pathway regulation network

Using multiple databases, miRNA-target relationships for core genes and lncRNA–miRNA interactions were predicted, constructing a ceRNA regulatory network. The visualization of the lncRNA–miRNA–mRNA–pathway network was further performed using Cytoscape.

### Gene set variation analysis

Using the GSVA package (version 2.2.0) in R, we performed GSVA analysis to assess pathway-level activity and identify differences in biological functions across molecular clusters, thereby elucidating the functional roles of the core genes. Specifically, GSVA was conducted using the Molecular Signatures Database (MSigDB v7.5.1) gene set collection, with the KEGG canonical pathways gene set (c2.cp.kegg.Hs.symbols.gmt) serving as the reference. GSVA enrichment scores for KEGG pathways were then computed across samples to systematically characterize gene functions and the underlying biological processes.

### Immune cell infiltration analysis

The CIBERSORT algorithm was applied to estimate the relative infiltration proportions of 22 immune cell types. Specifically, immune-cell composition was compared between 17 adjacent normal tissues and 87 treatment samples in GSE86374. Differences in immune-cell infiltration across the two groups were assessed using the Wilcoxon signed-rank test (*P* < 0.05). Furthermore, using Spearman’s correlation, we evaluated relationships between core target gene expression and infiltration levels of specific immune cells, thereby revealing their potential roles in immune regulation within the tumor microenvironment.

### Molecular docking

Crystal structures of the core target proteins were retrieved from the RCSB Protein Data Bank (https://www.rcsb.org/). Protein structures were preprocessed in PyMOL by removing crystallographic water molecules and separating co-crystallized ligands. The prepared structures were then imported into AutoDockTools v1.5.7 for the addition of polar hydrogens, assignment of Gasteiger charges, and merging of nonpolar hydrogens, followed by conversion to PDBQT format. Molecular docking was conducted using AutoDock Vina *via* command-line execution. The docking search space was defined as a grid box with a fixed size of 40  ×  40  × 40 Å (grid size_x/y/z) and a grid spacing of 1 Å. The grid box centers were set to (*x* = 14.845, *y* = 32.473, *z* = 7.132) for AURKA (PDB ID: 5DPV), (*x* = 75.938, *y* = 34.107, z = −74.891) for INHBA (PDB ID: 5HLY), (x = −6.976, *y* = 13.141, *z* = 27.250) for KIF11 (PDB ID: 6TRL), and (*x* = 15.764, *y* = 12.376, *z* = 16.339) for NEK2 (PDB ID: 2XNM). AutoDock Vina parameters were set as follows: num_modes = 30 and energy_range = 5 (Table S1). The final visualization of the docking results was performed using PyMOL.

### Molecular dynamics simulation and binding stability assessment

To assess the binding stability of BaP–target protein complexes, 100-ns MD simulations were conducted in the GROMACS software package (Sinelnikova & Spoel, 2021). The simulation system was constructed as follows: the protein was described with the AMBER14SB force field, while the GAFF force field was applied to the small-molecule ligand, with its partial atomic charges assigned using the Bond Charge Correction (BCC) method. The complex was solvated in a dodecahedral water box using the TIP3P water model. Counterions were added to neutralize the system.

The simulation workflow comprised three sequential phases: energy minimization, equilibration, and production. Energy minimization was conducted with the steepest descent algorithm (maximum steps = 50,000, step size = 0.01 nm). Subsequently, the system was equilibrated under the NVT ensemble for 200 ps, followed by the NPT ensemble for another 200 ps. During equilibration, a time step of 2 fs was used, and all bonds involving hydrogen atoms were constrained using the LINCS algorithm. The system temperature was maintained at 298 K using the Nosé–Hoover thermostat, and the pressure was regulated at 1 bar using the Parrinello–Rahman barostat. Long-range electrostatic interactions were treated using the Particle Mesh Ewald (PME) method, with a cutoff radius of 1.2 nm applied for both van der Waals and Coulombic interactions. Following equilibration, a 100 ns production molecular dynamics simulation was conducted, with trajectory frames saved every 10 ps. The trajectory from the production run was used for stability analysis, including calculations of the root mean square deviation (RMSD), root mean square fluctuation (RMSF), radius of gyration (Rg), and solvent accessible surface area (SASA). In addition, binding free energies were estimated using the MM/PBSA approach implemented in gmx_MMPBSA. Frames for the MM/PBSA calculations were extracted from the equilibrated portion of the production MD trajectory (98–100 ns). A total of 11 frames were analyzed, sampled at a constant interval of 0.2 ns. This sampling strategy was used to ensure reproducible and uniform coverage of the stable binding conformation while avoiding the use of highly adjacent, strongly correlated frames. The solute and solvent dielectric constants were set to *ɛ*_in = 2 and *ɛ*_out = 80. Poisson–Boltzmann calculations were performed with a grid spacing of 0.5 Å and an ionic strength of 0.15 M. The solvent probe radius was set to 1.4 Å, and nonpolar solvation energies were estimated using the SASA model. Entropic contributions (–TΔS) were estimated by normal mode analysis (NMA) using the nmode module in gmx_MMPBSA at 298 K. To ensure consistency between the enthalpic and entropic terms, NMA was performed on the same frames used for the MM/PBSA calculations, and no additional subsampling was applied. Before NMA, each selected snapshot was energy-minimized to obtain a local minimum suitable for Hessian matrix construction. Vibrational entropy was then calculated from the eigenvalues of the diagonalized Hessian matrix, whereas translational and rotational entropy terms were evaluated using standard rigid-body statistical mechanical formulations. The use of 11 evenly spaced frames was chosen as a practical compromise between conformational sampling and computational feasibility, because NMA requires energy minimization and Hessian diagonalization for each frame and is therefore substantially more computationally demanding than the MM/PBSA enthalpic calculations.

## Results

### Screening and identification of potential targets associated with BaP exposure and BC

An overview of the study workflow is presented in Fig. S1. Potential targets were screened by integrating multiple databases. Integration of the ChEMBL, PharmMapper, and SEA databases yielded 395 potential BaP targets. Concurrently, 5,472 BC-associated genes were collected from GeneCards and OMIM. Subsequently, Venn online analysis was performed to integrate these target sets, and 216 common targets were identified that were shared by BaP and BC (Table S2). The results indicate that these genes may function as pivotal mediators in BaP-promoted BC progression.

### Enrichment analyses of GO terms and KEGG pathways

**Figure 1:**
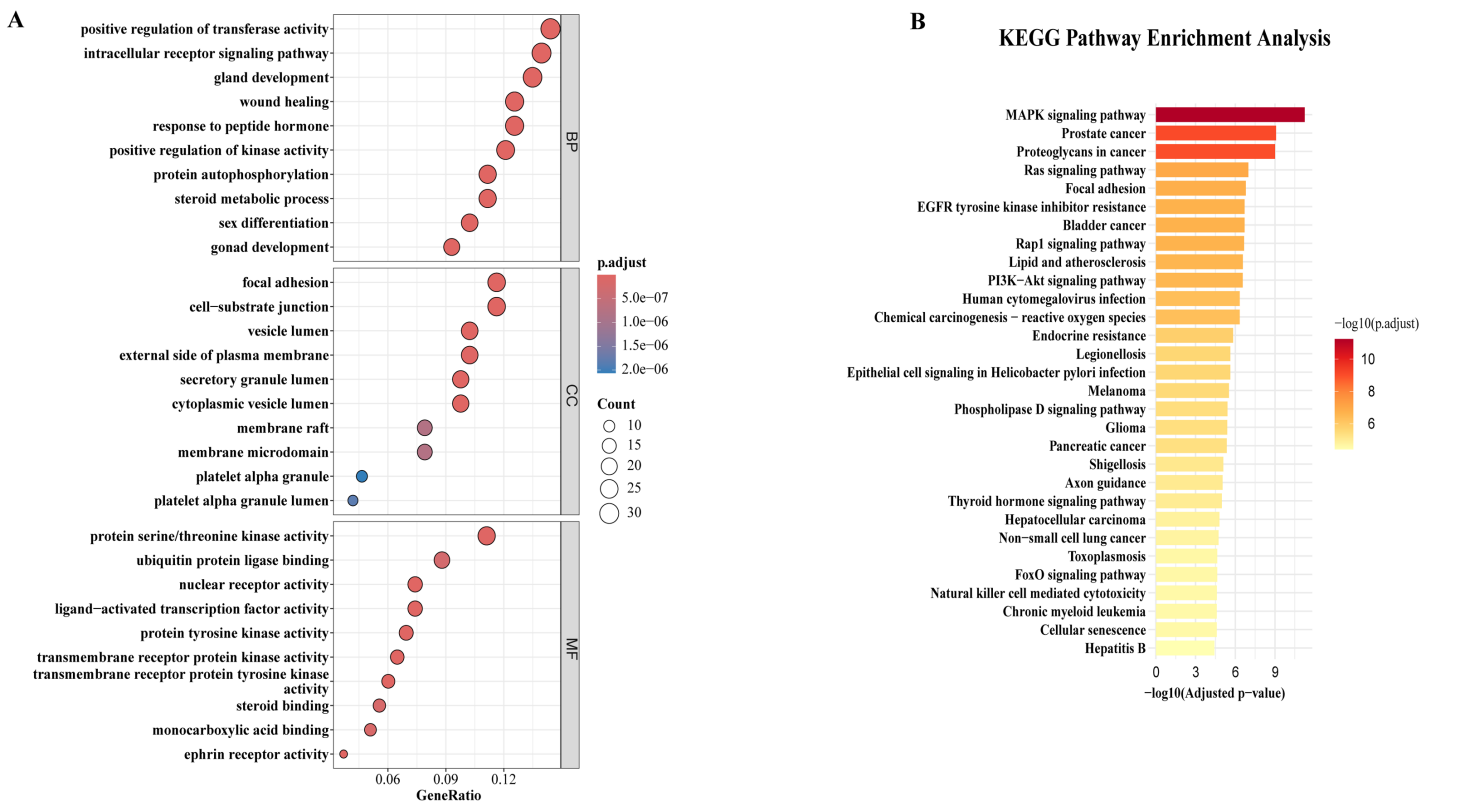
GO functional analysis and KEGG pathway analysis between BaP exposure and BC. (A) GO functional analysis bubble chart. (B) KEGG analysis histogram, ordered by *P*-value from smallest to largest.

To systematically analyze the biological roles and regulatory pathways of the common targets associated with BaP exposure and BC, GO term annotation and KEGG pathway enrichment of 216 putative targets were carried out *via* the DAVID database (Table S3). GO analysis identified 597 significantly enriched terms (*P* < 0.05), comprising 260 BP, 189 CC, and 148 MF entries. The top 10 most significant terms in each category, ranked by *P*-value, were visualized (Fig. 1A). Among these, BP terms were primarily enriched in processes such as protein autophosphorylation, intracellular receptor signaling pathways, and positive regulation of transferase activity; CC terms were primarily associated with focal adhesions and cell–matrix junctions; and MF terms were associated with nuclear receptor activity and ligand-activated transcription factor activity.

Subsequent KEGG pathway analysis revealed that these targets were predominantly enriched in several signaling pathways closely associated with tumorigenesis and progression, including the MAPK signaling pathway, the Ras signaling pathway, and EGFR tyrosine kinase inhibitor resistance. The top 20 pathways with the smallest *p*-values were chosen for visualization (Fig. 1B). This preliminary finding suggested that BaP could participate in the pathogenesis of BC by regulating the aforementioned pathways.

### The core target screening of machine learning algorithms

**Figure 2:**
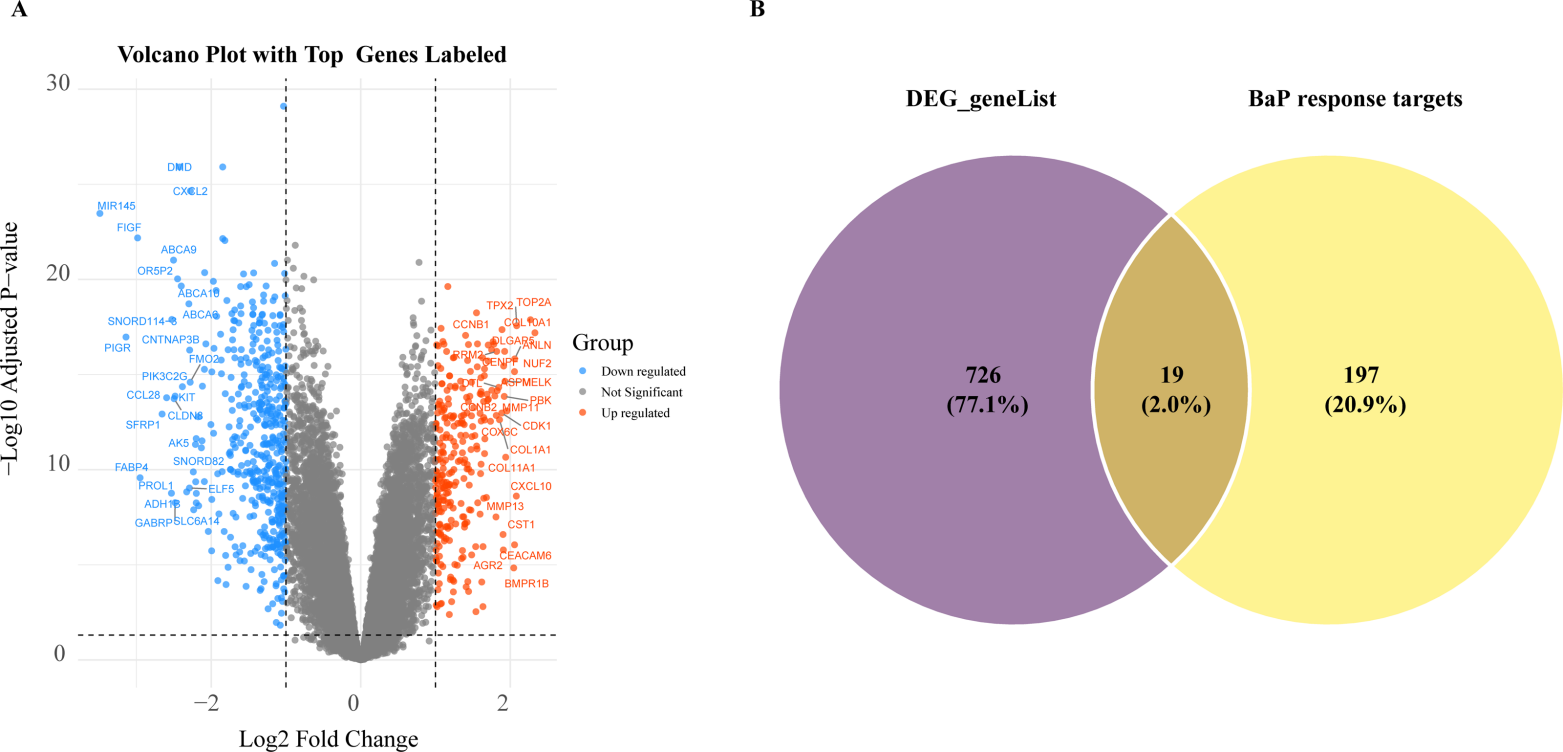
Molecular characterization of BaP exposure and BC. (A) Volcano plot analysis of differentially expressed genes; significantly upregulated (red) and downregulated (blue) genes are highlighted, and each point represents a gene. (B) Venn diagram illustrating overlapping genes between BC-associated differentially expressed genes and BaP response targets.

Differential expression analysis of the GSE86374 dataset revealed 745 DEGs significantly associated with BC, including 272 upregulated and 473 downregulated genes (Fig. 2A, Table S4). Intersection of these DEGs with 216 BaP-related targets yielded 19 overlapping genes (Fig. 2B), implying pivotal regulatory involvement in BC initiation and progression.

**Figure 3:**
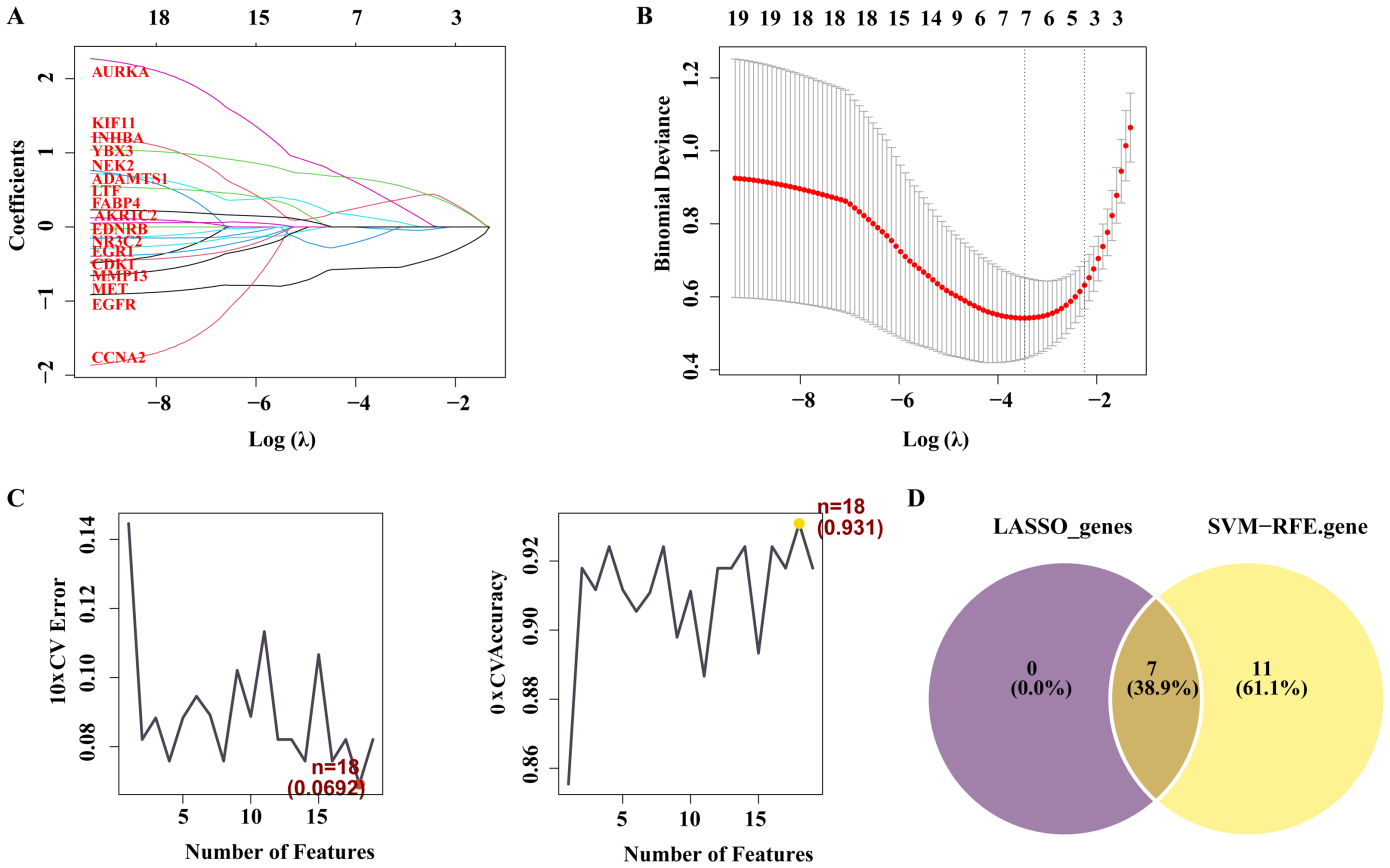
Machine-learning algorithms for key gene screening. (A–B) Key genes were screened using the LASSO regression algorithm. (C) Key genes were screened using the SVM-RFE algorithm. (D) A Venn diagram of the intersection of key genes identified by the LASSO and SVM-RFE algorithms is shown.

To further identify diagnostic core genes for BC, LASSO logistic regression and SVM-RFE were applied for dimensionality reduction and feature selection among 19 candidate genes. Seven key genes were identified by LASSO (Figs. 3A and 3B), whereas 18 feature genes were selected using the SVM-RFE algorithm (Fig. 3C, Table S5). By intersecting the genes identified by both algorithms, seven BC biomarkers with high diagnostic potential were ultimately determined, namely EGFR, KIF11, INHBA, NR3C2, NEK2, AURKA, and YBX3 (Fig. 3D).

### Validation of diagnostic markers

**Figure 4:**
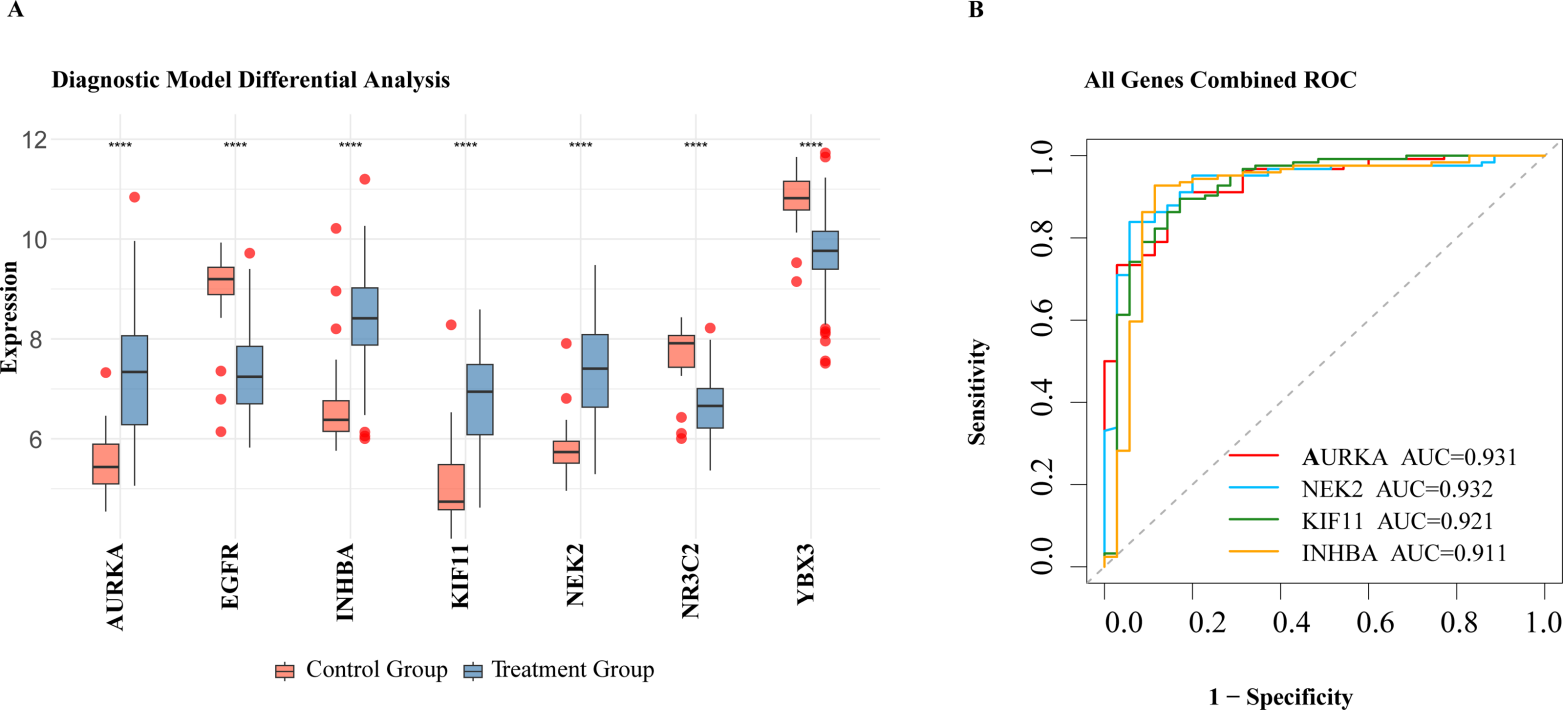
High expression of key gene markers for BC diagnosis and validation of their diagnostic performance. (A) Expression patterns of genes associated with BaP exposure and breast cancer, ****P < 0.0001. (B) To appraise diagnostic performance, ROC curves were constructed for the selected genes.

In the GSE86374 validation cohort, the expression levels of AURKA, NEK2, KIF11, and INHBA were significantly higher in breast cancer tissues than in normal adjacent breast tissues (*P* < 0.0001) (Fig. 4A, Fig. S2), whereas EGFR, NR3C2, and YBX3 showed a significant trend toward downregulation. In this study, we prioritized the upregulated genes for downstream analyses: (i) our main objective was to identify overexpressed biomarkers with practical diagnostic utility in BC, (ii) upregulated targets are generally more amenable to therapeutic intervention (*e.g.*, inhibition), and (iii) these four genes exhibited strong diagnostic performance in ROC analyses. Specifically, ROC curves showed that NEK2 (AUC = 0.932), AURKA (AUC = 0.931), KIF11 (AUC = 0.921), and INHBA (AUC = 0.911) achieved excellent discriminative ability between breast cancer and adjacent normal tissues (Fig. 4B). Taken together, these findings highlight the substantial clinical potential of these upregulated gene biomarkers for the early diagnosis of BC.

### External dataset validation

**Figure 5:**
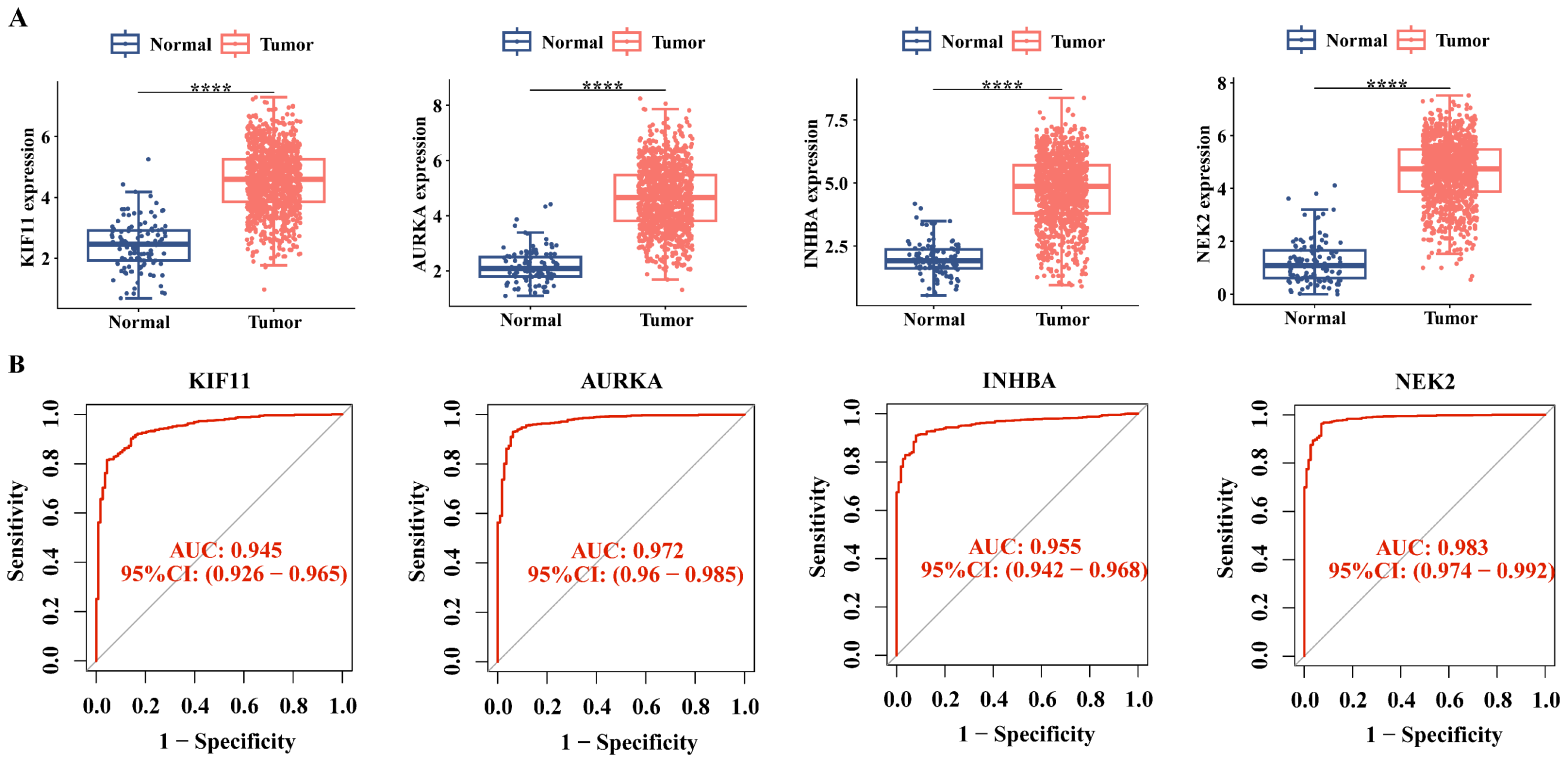
External validation of four hub genes in the TCGA-BRCA cohort. (A) Box plots showing the expression levels of KIF11, AURKA, INHBA, and NEK2 in breast cancer (Tumor) tissues compared with adjacent normal (Normal) breast tissues; ****P < 0.0001. (B) ROC curves evaluating the diagnostic performance of each gene for discriminating tumors from normal tissues in TCGA-BRCA.

To further confirm the clinical relevance of the core genes in a larger cohort, we conducted external validation using the independent TCGA-BRCA dataset. As shown in Fig. 5A and Table S6, the expression levels of AURKA, NEK2, KIF11, and INHBA were significantly upregulated in BC tissues compared to adjacent normal tissues (all *P* < 0.001), which was highly consistent with our findings in the GSE86374 dataset. ROC curve analysis (Fig. 5B) further demonstrated the strong diagnostic ability of these four genes: AURKA (AUC = 0.972), NEK2 (AUC = 0.983), KIF11 (AUC = 0.945), and INHBA (AUC = 0.955). These results, reproduced in an independent and large-scale cohort, strongly support the reliability of these four genes as potential biomarkers for BC.

### Regulatory network of lncRNA–miRNA–mRNA–Pathways

**Figure 6:**
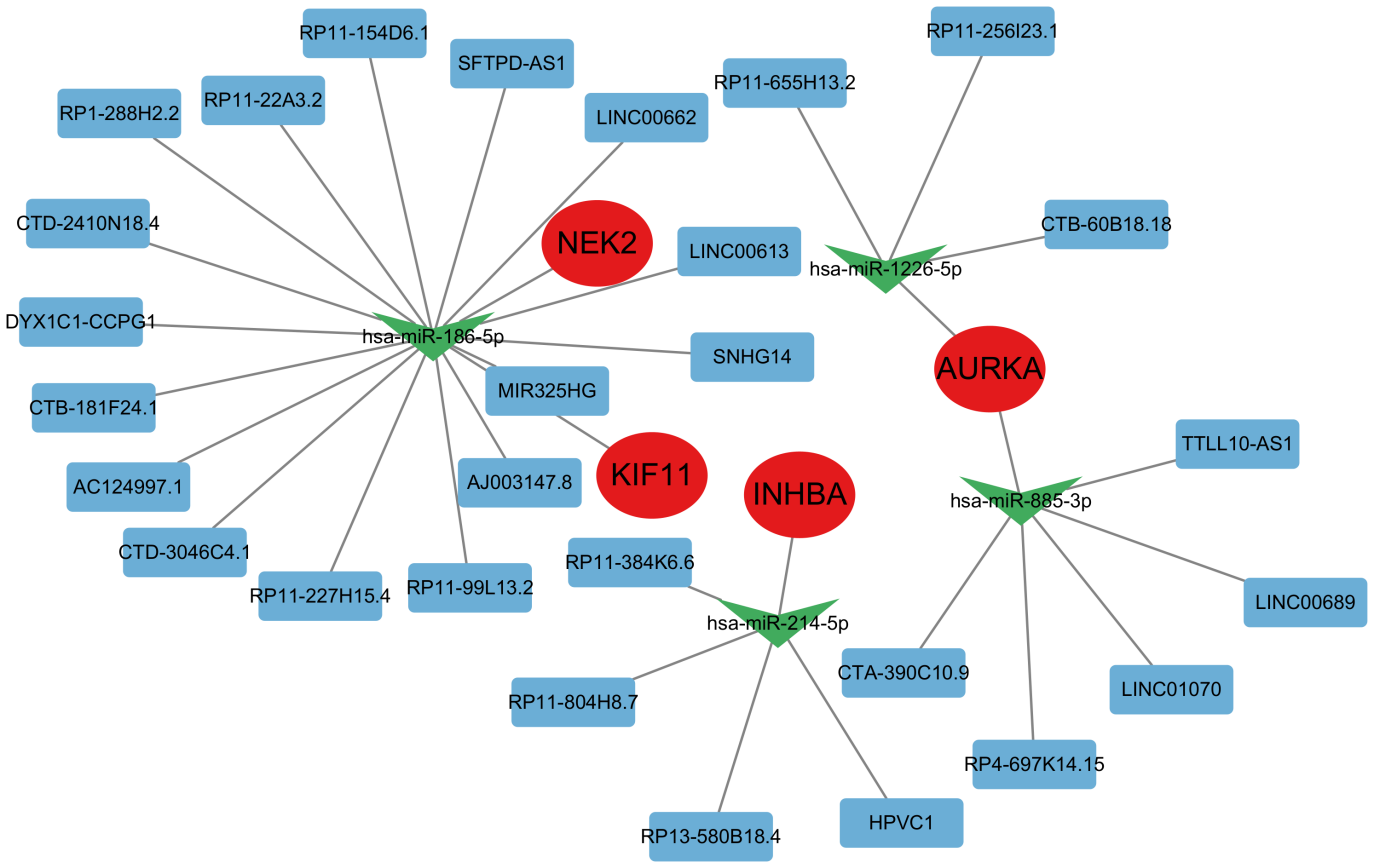
The lncRNA-miRNA-mRNA-pathway regulatory network diagram. Circles represent mRNA, rectangles represent pathways, diamonds represent lncRNA, and V-shapes represent miRNA.

Upstream miRNA prediction was conducted for the four candidate diagnostic genes (based on the miRanda, miRTarBase, miRDB, and TargetScan databases), yielding a total of 13 interacting miRNAs. Further analysis, conducted using the spongeScan database, identified 28 miRNA-lncRNA interaction pairs. By integrating core genes, annotated pathways, and the aforementioned interactions, an integrated lncRNA–miRNA–mRNA–pathway ceRNA regulatory network comprising 40 nodes (four mRNAs, 13 miRNAs, 28 lncRNAs, and four pathways) was constructed using Cytoscape (Fig. 6).

### Analysis of GSVA enrichment and immune cell infiltration

**Figure 7:**
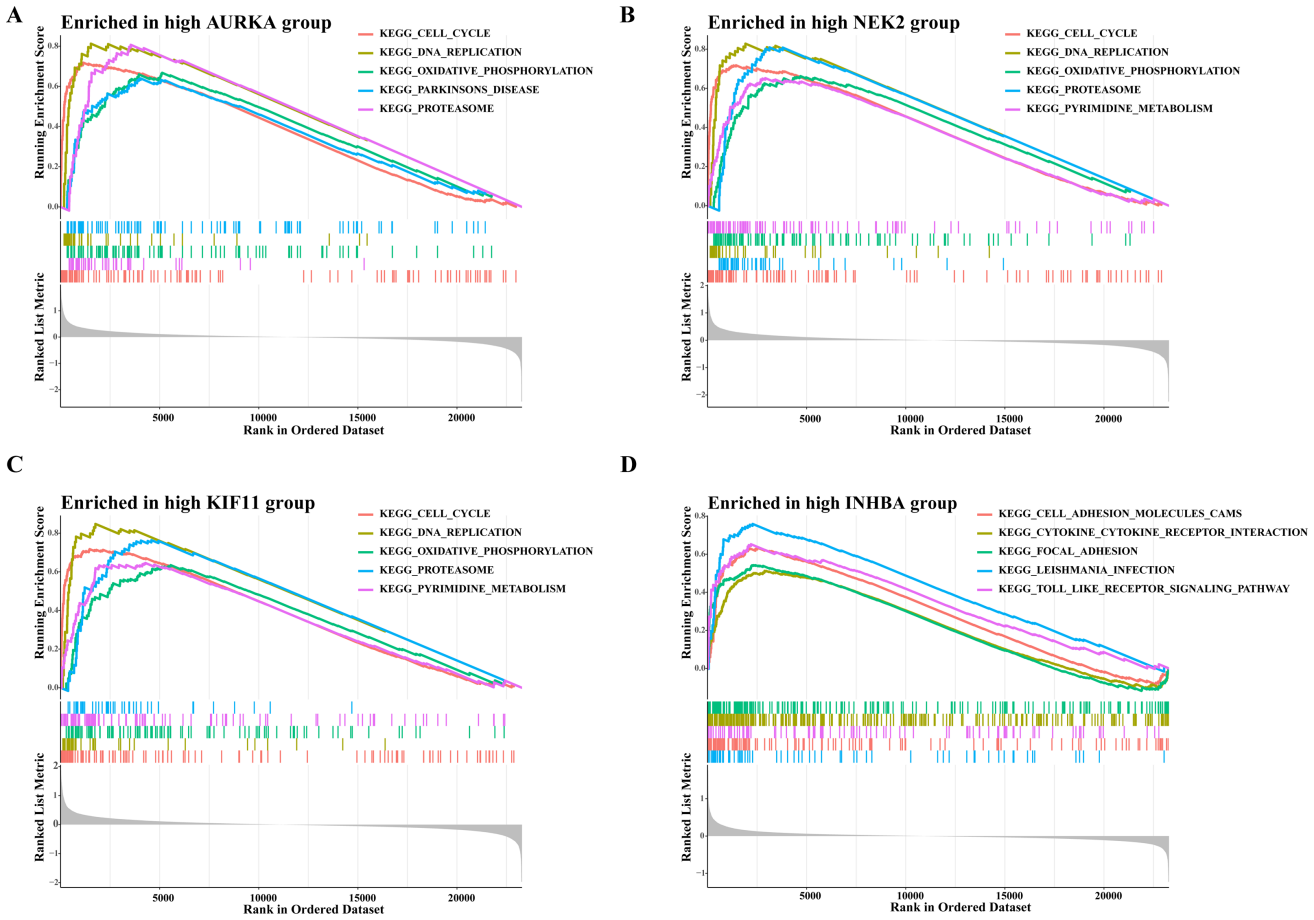
Gene set variation analysis (GSVA) of BaP–BC signature genes. (A) GSEA results for AURKA are shown. (B) GSEA results for NEK2 are shown. (C) GSEA results for KIF11 are shown. (D) GSEA results for INHBA are shown.

To further explore the biological functions of BaP in BC, GSVA was conducted. Results revealed that four core genes—AURKA, NEK2, KIF11, and INHBA—are markedly enriched in pathways related to cell-cycle control, DNA replication, oxidative phosphorylation, and CAMs (Fig. 7). These pathways are pivotal in BC initiation and progression, acting not only through direct oncogenic mechanisms such as cell cycle dysregulation and DNA replication abnormalities but also indirectly by promoting tumor formation and progression through the regulation of cell migration and activation of the immune microenvironment.

**Figure 8:**
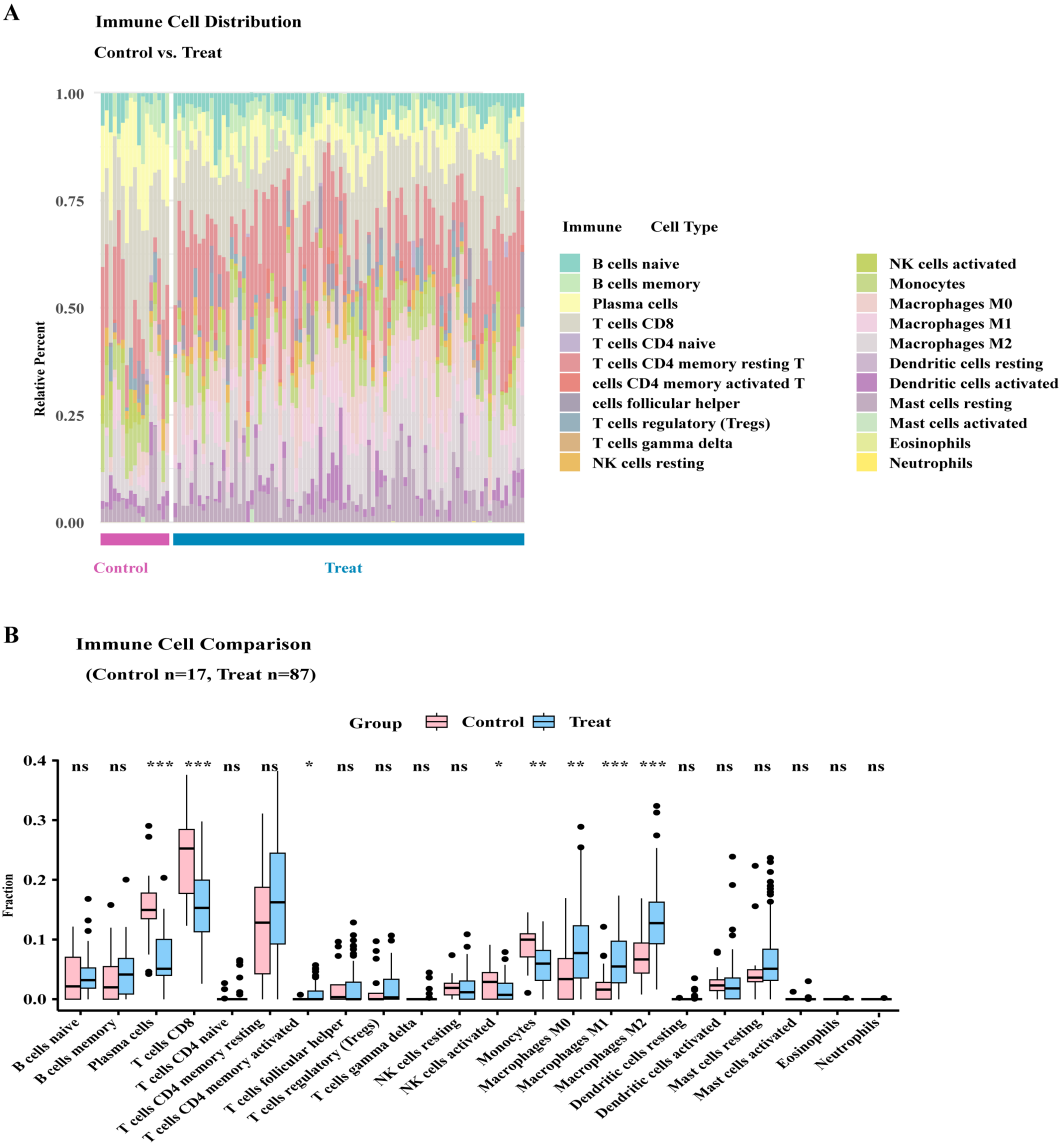
Landscape of immune cell infiltration in breast cancer. (A) Box plots illustrated the distribution of 22 immune cell subtypes in BC *versus* normal samples. (B) Significant differences existed in immune infiltration levels between BC and normal control groups (*P* < 0.05).

**Figure 9:**
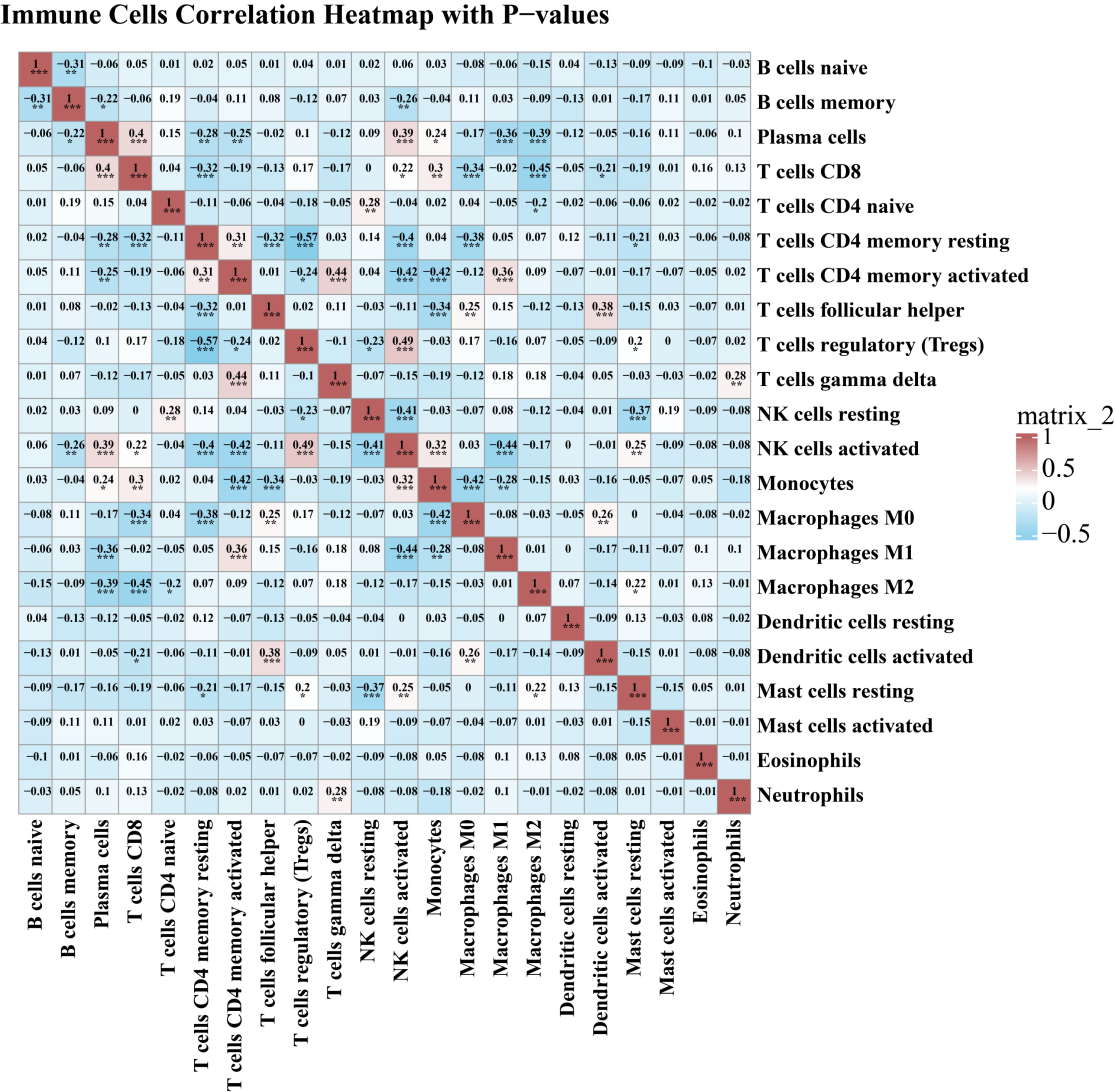
Analysis of immune cell correlations. A heatmap of correlations among 22 immune cell populations is presented; Positive (red) and negative (blue) correlations are distinguished, with white indicating no meaningful correlation.

Infiltration patterns of 22 immune cell populations in BC and adjacent normal tissues were evaluated through the CIBERSORT algorithm. Significant differences were identified in the infiltration proportions of activated CD4^+^ memory T cells and multiple macrophage subtypes (M0/M1/M2) (Figs. 8A and 8B). Correlation analysis among immune cells revealed positive associations (Fig. 9) between M1 macrophages and activated CD4^+^ memory T cells, between neutrophils and γδ T cells, and between CD4^+^ memory T cells and Tfh cells. Conversely, negative associations were observed between regulatory T (Treg) cells and resting CD4^+^ memory T cells, along with those between resting NK cells and CD8^+^ T cells.

**Figure 10:**
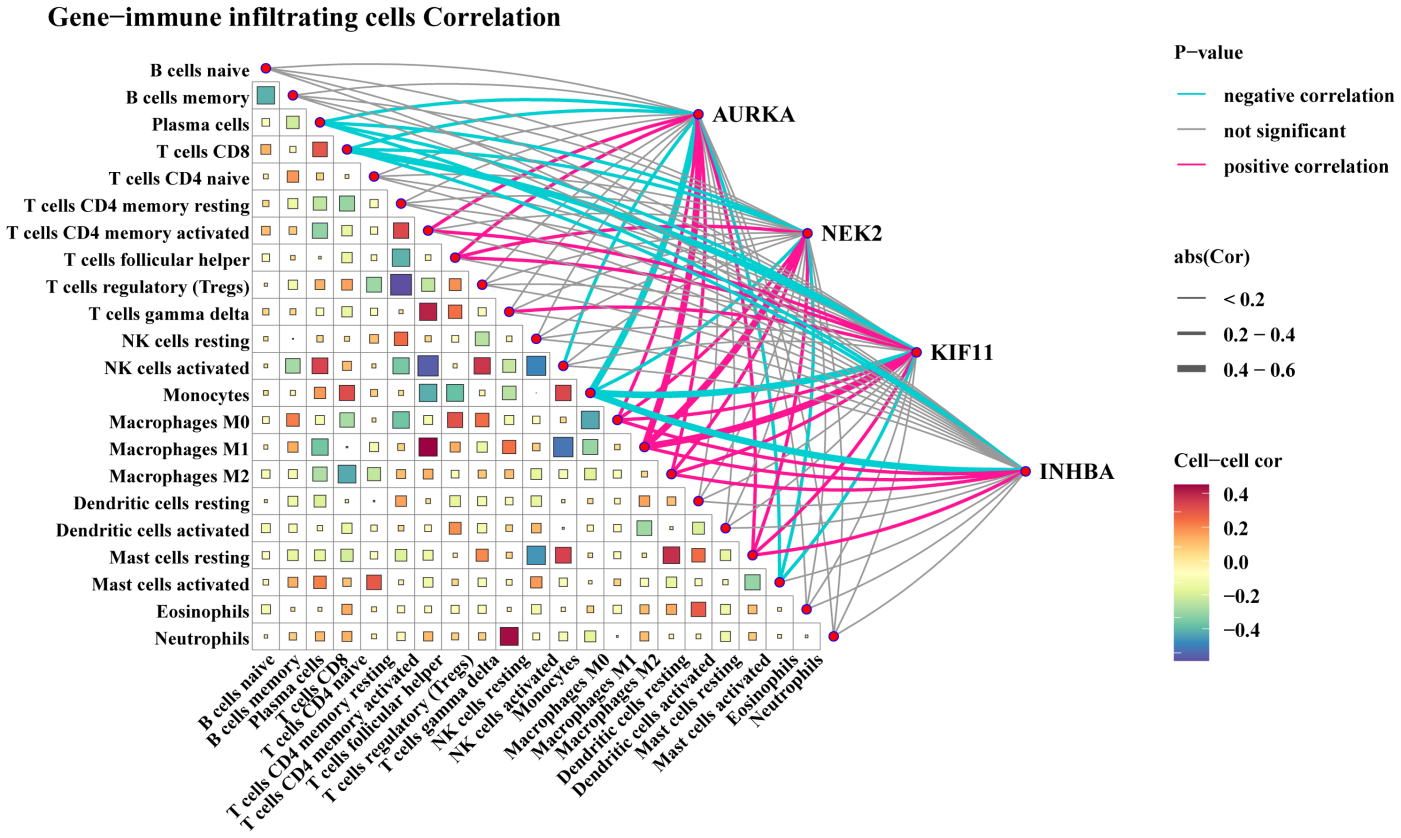
Correlation analysis between key genes and immune cell infiltration in breast cancer. Correlation analyses between the expression levels of AURKA, NEK2, KIF11, and INHBA and immune cell infiltration in BC samples are shown.

Through additional analysis, we examined the correlations between immune cells and the four key gene targets (Fig. 10). AURKA, NEK2, KIF11, and INHBA were positively correlated with CD4^+^ memory T cells, Tfh cells, and M0/M1/M2 macrophages, whereas negative correlations were observed with plasma cells, CD8^+^ T cells, and activated NK cells. These findings suggest that these genes may be associated with immune cell infiltration patterns in the BC tumor microenvironment, potentially influencing immune evasion processes.

### Molecular docking verification

To investigate how BaP engages with the principal target proteins, molecular docking methods were employed, with binding energy parameters used as the criterion for successful docking. A higher absolute value of the binding energy parameter is associated with stronger ligand–receptor binding stability. Docking analyses were conducted using AutoDock and PyMOL software. In general, values below −5 kcal/mol are considered indicative of significant binding activity of the ligand to the protein complex.

**Figure 11:**
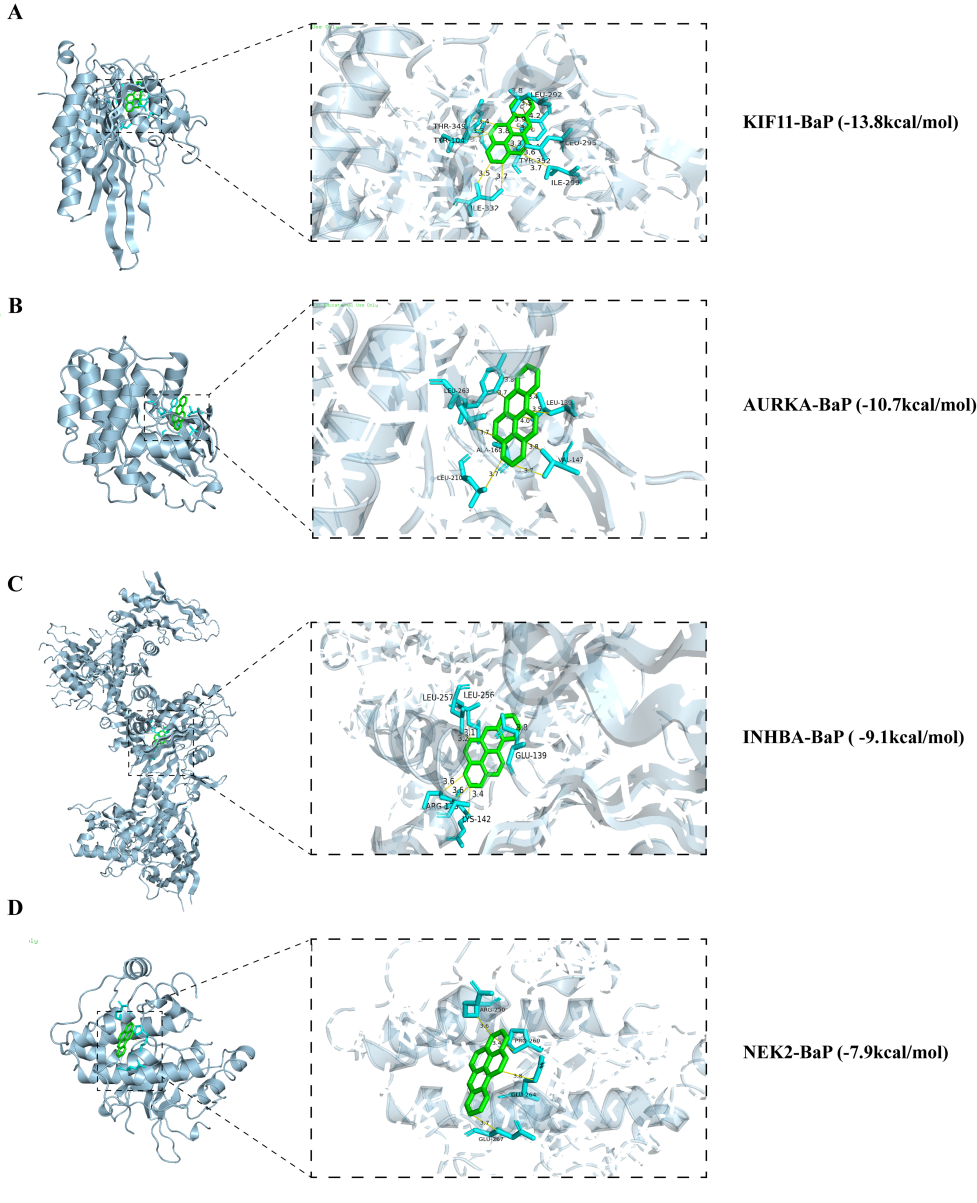
Molecular docking of key targets. (A) KIF11 and BaP. (B) AURKA and BaP. (C) INHBA and BaP. (D) NEK2 and BaP.

The docking analysis demonstrated that BaP exhibited appreciable binding affinity toward all four core target proteins (Fig. 11). Specifically, the most favorable binding energy was obtained for KIF11 (−13.8 kcal/mol), followed by AURKA (−10.7 kcal/mol), INHBA (−9.1 kcal/mol), and NEK2 (−7.9 kcal/mol). To contextualize whether these docking scores may reflect biologically meaningful interactions, we further compared BaP with established inhibitors (positive-control ligands) reported for each target, which were curated from the literature (Xi et al., 2017; Thankamony et al., 2020; Chen et al., 2021; Yu et al., 2021; Kou et al., 2025): AURKA–Alisertib (−9.2 kcal/mol), KIF11–Ispinesib (−6.93 kcal/mol), INHBA–SB-431542 (−8.46 kcal/mol), and NEK2–MBM-55S (−7.70 kcal/mol). These values correspond to the best-scoring poses among the generated conformations for each protein. Detailed docking scores, along with RMSD values for all poses, are provided in Table S7. Under the same docking protocol, the predicted binding energies of BaP were within a comparable range to those of the corresponding reference inhibitors across these targets, supporting the plausibility of stable pocket engagement by BaP. Collectively, these results suggest that BaP may directly interact with multiple hub proteins implicated in BC progression, thereby providing a structural rationale for its potential tumor-promoting activity. However, docking scores are not direct measures of inhibitory potency, and further cellular validation is required to substantiate these predicted interactions.

### Analysis of molecular dynamics simulation results

To further evaluate the conformational stability of BaP in complex with four core targets, we conducted three independent MD simulations for the aforementioned complexes. Each simulation lasted from 0 to 100 ns, and random initial velocities were set to verify the consistency of protein-ligand interactions under different initial dynamic conditions.

**Figure 12:**
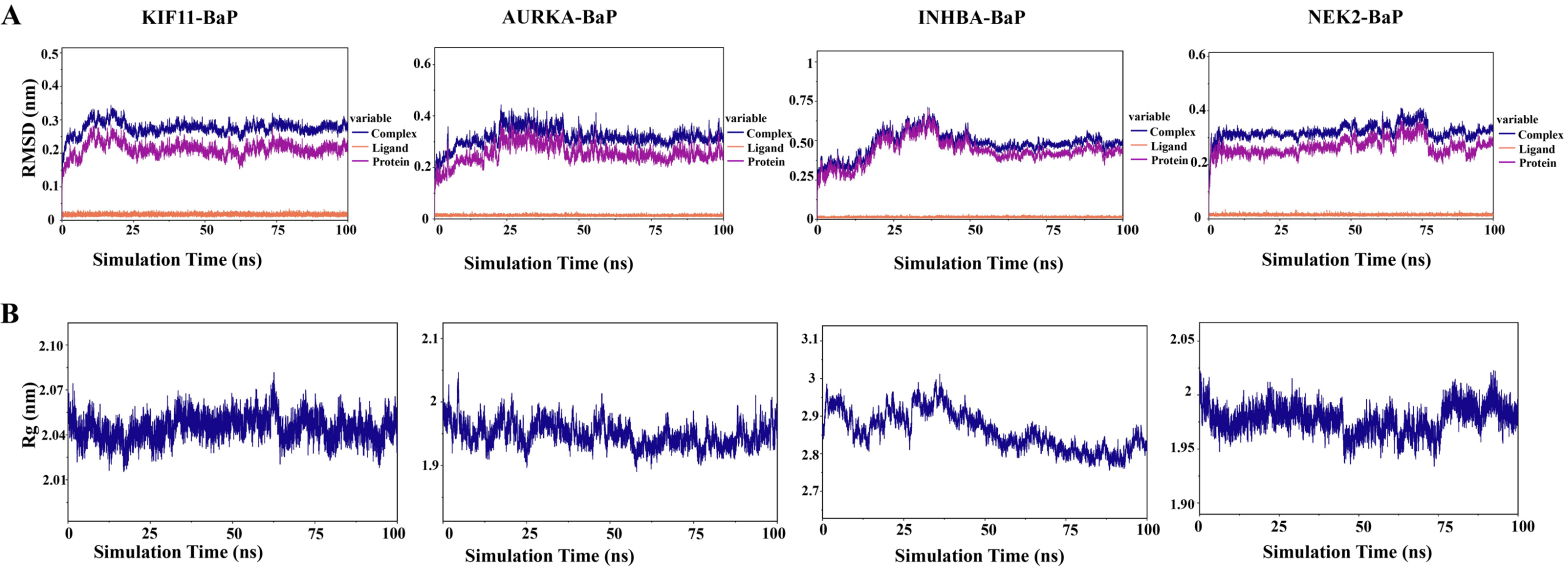
Conformational stability and dynamic characteristics of the turning radius of the complexes of KIF11, AURKA, INHBA, and NEK2 with BaP. (A) RMSD evolution curve. (B) RG curve.

MD simulations were performed to evaluate the structural integrity of four protein-ligand complexes: KIF11-BaP, AURKA-BaP, INHBA-BaP, and NEK2-BaP. Results from three independent replicate runs, initiated with different random initial velocities, collectively demonstrated robust structural stability across all four systems. RMSD analysis was conducted for each system to assess conformational deviation over time (Fig. 12, Figs. S3, S5). The RMSD of the KIF11-BaP complex was found to fluctuate minimally, consistently staying within the 0.2−0.35 nm range, which indicates long-term conformational stability. For the AURKA-BaP complex, the RMSD was observed to range between 0.15–0.45 nm with smooth fluctuations, reflecting good conformational consistency and simulation reproducibility. The INHBA-BaP complex generally maintained its RMSD within the 0.4–1.0 nm range, suggesting that fundamental structural stability was preserved. However, a transient RMSD increase was noted during the 25–40 ns interval of the third simulation replicate, reaching a peak of approximately 3 nm. This fluctuation was determined to be the result of a brief, localized conformational disturbance, after which the RMSD rapidly returned to the stable range near 1.0 nm (Fig. S5). The RMSD of the NEK2-BaP complex fluctuated stably within the 0.3–0.4 nm range. During the first 50 ns, it remained largely between 0.15 and 0.25 nm, then rose slightly to approximately 0.3 nm after 50 ns and remained steady thereafter.

Rg analysis was employed to rigorously assess the overall structural compactness of the protein-ligand complexes. Results consistently indicated that the Rg values for all systems rapidly converged to a stable equilibrium during the initial 0–100 ns simulation period. This rapid stabilization suggests that the proteins quickly adopted their compact, native-like folded conformations following ligand binding (Fig. 12, Figs. S3, S5). The Rg of the KIF11-BaP complex consistently fluctuated within the 2.01–2.08 nm range, with no discernible trend being observed. The Rg of the AURKA-BaP complex oscillated between 1.85 and 2.0 nm, demonstrating no evidence of significant structural expansion (loosening) or aggregation. The Rg of the INHBA-BaP complex was primarily distributed within the 2.8–3.1 nm range, exhibiting relatively smooth conformational fluctuations, which confirms the strong overall structural stability of the complex. However, during the third simulation replicate, a transient Rg increase was recorded between 30 and 40 ns, reaching a peak of 3.6 nm. This spike represented a temporary conformational perturbation, followed by the rapid subsequent return of Rg to the stable range. This characteristic suggests that the complex possesses robust conformational self-recovery capability (Fig. S5). The Rg value of the NEK2-BaP complex fluctuated between 1.95 and 2.05 nm, showing a slight, temporary decrease near 50 ns before quickly recovering its initial stable state.

**Figure 13:**
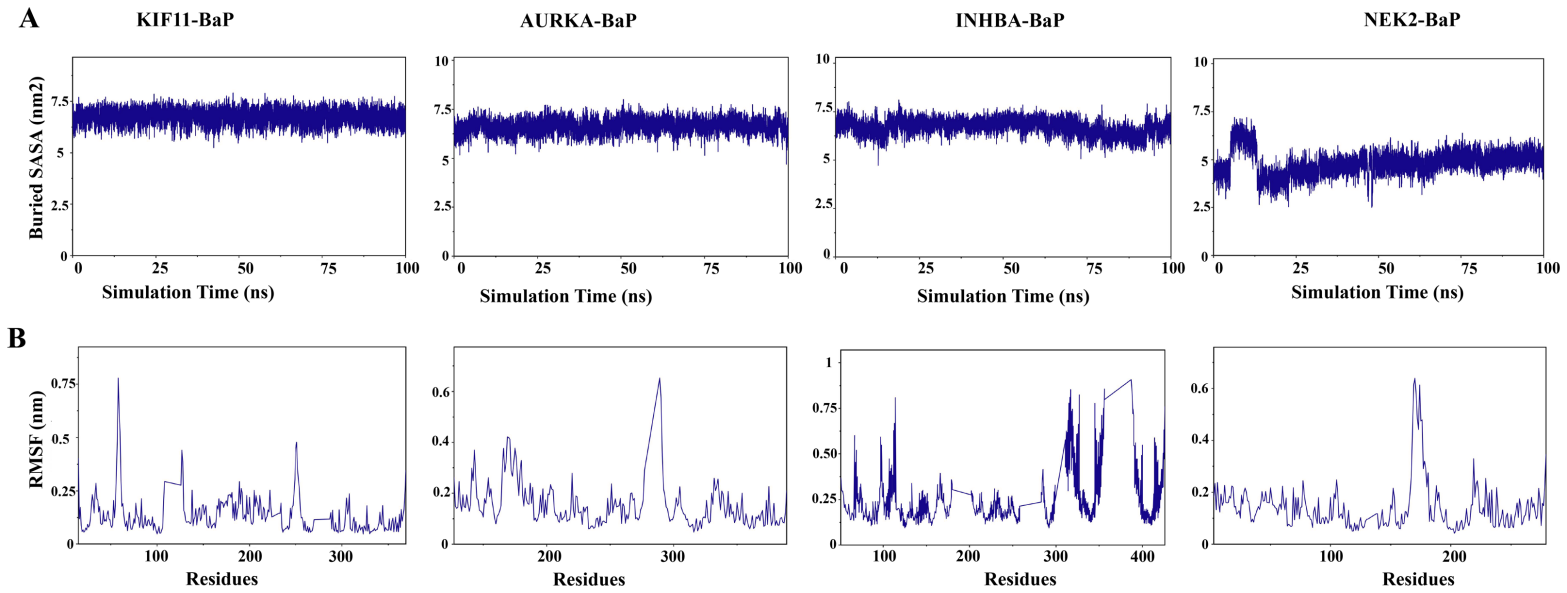
Analysis of solvent-accessible surface area and residue fluctuation of the complexes of KIF11, AURKA, INHBA, and NEK2 with BaP. (A) SASA fluctuation curve. (B) Amino acid residue fluctuations.

SASA analysis was utilized to evaluate the interaction characteristics between the complexes and solvent molecules, thereby reflecting the stability of their surface structures. The results consistently demonstrated that the SASA values for each complex remained highly stable throughout the entire 0–100 ns simulation period. Fluctuations were concentrated within a narrow range of 0–7.5 nm^2^ showing no significant fluctuations or trend-like changes (Fig. 13, Figs. S4, S6). This outcome suggests that following ligand binding to the target protein, the hydrophilic-hydrophobic distribution on the protein surface rapidly stabilizes, leading to the establishment of a robust dynamic equilibrium with the surrounding solvent molecules.

RMSF analysis was utilized to further evaluate the local flexibility of individual amino acid residues within the receptor protein of each complex. Key residues that significantly contribute to the binding energy in the KIF11-BaP complex (*e.g.*, 104TYR and 292LEU) consistently exhibited RMSF values below 0.2 nm. This result is indicative of strong local conformational constraints in these critical regions (Fig. 13, Figs. S4, S6). The corresponding RMSF values for key residues in the AURKA-BaP complex (*e.g.*, 139LEU) were also observed to be within the low-fluctuation range. Residues contributing to strong binding energy in the INHBA-BaP complex (*e.g.*, 137HIS and 239ASP) were found to have RMSF values that fall within the low-fluctuation range of 0–0.25 nm, signifying high structural rigidity in these specific areas. Notably, during the third independent simulation replicate, the residue range 300–400 exhibited a significantly high fluctuation (RMSF ≈ 3 nm), whereas the RMSF values in all other regions remained below 1 nm. This pattern suggests relatively pronounced local flexibility in the C-terminal region of the INHBA protein, while the core functional domain (the low-RMSF region) maintains strong conformational rigidity (Fig. S6). The RMSF values for key residues in the NEK2-BaP complex (*e.g.*, 247ILE) were also consistently below 0.25 nm, further supporting the conclusions regarding the structural stability of the binding sites.

To evaluate the binding strength among different complexes, the MM/PBSA method was used to calculate the binding free energy. Each system underwent three independent repetitions of experiments, and the final results were averaged, as presented in Table 1. The binding free energies between BaP and the four targets (KIF11, AURKA, INHBA, and NEK2) ranged from −14.648 to −34.817 kcal/mol. Negative ΔGbind values indicate spontaneous binding, with more negative values corresponding to stronger binding affinity. Among the four targets, KIF11 exhibited the most negative ΔGbind value (−34.817 kcal/mol), indicating the strongest binding and highest complex stability. This strong binding is primarily driven by van der Waals interactions, with hydrophobic interactions playing a secondary role, likely due to the high shape complementarity between the rigid, planar BaP molecule and the deep, highly hydrophobic binding pocket of KIF11.

**Table 1:** Distribution of energy contributions from various interacting components to the binding free energy of the complex (kcal/mol).

**Protein-Ligand**	**ΔEvdw**	**ΔEele**	**ΔEpol**	**ΔEnonpol**	**ΔEMMPBSA**	**-TΔs**	**ΔGbind**
KIF11-BaP	−40.496 ± 0.041	−0.580 ± 0.009	8.764 ± 0.081	−4.202 ± 0.005	−36.514 ± 0.136	1.697 ± 0.216	−34.817 ± 0.254
AURKA-BaP	−34.370 ± 0.183	−0.524 ± 0.035	11.737 ± 0.136	−4.206 ± 0.013	−27.363 ± 0.231	2.179 ± 0.263	−25.184 ± 0.347
INHBA-BaP	−21.798 ± 0.299	−0.342 ± 0.040	7.496 ± 0.080	−3.427 ± 0.032	−18.071 ± 0.160	3.423 ± 0.461	−14.648 ± 0.488
NEK2-BaP	−27.496 ± 0.141	−2.099 ± 0.021	12.768 ± 0.071	−3.335 ± 0.006	−20.162 ± 0.093	1.566 ± 0.107	−18.596 ± 0.142

**Notes.**

ΔEele Electrostatic interaction ΔEvdw Van der Waals interactions ΔEpol Polar solvation energy ΔEnonpol the nonpolar solvation energy ΔEMMPBSA ΔEvdw + ΔEele + ΔEpol + ΔEnonpol ΔGbind ΔEMMPBSA + (−TΔS)

**Figure 14:**
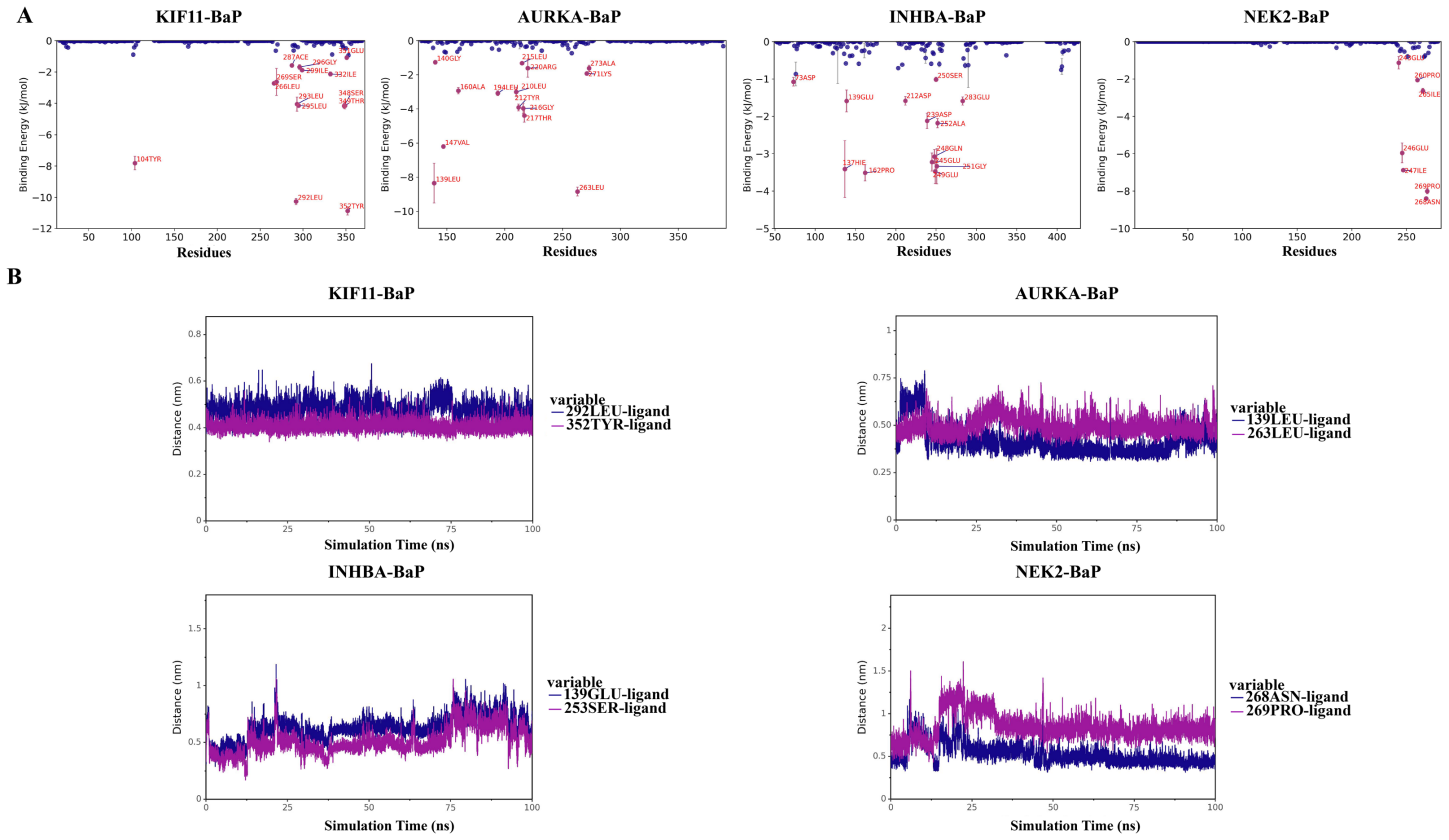
Key amino acid binding contributions and the distance between the ligand and the active site. (A) The contribution of key amino acids to the binding free energy. (B) Time-dependent minimum heavy-atom distances between the ligand and the side chains of the two key active-site residues during the 100 ns MD simulation.

To further elucidate the binding mechanism, a per-residue energy decomposition of the total binding free energy (ΔEMMPBSA) was performed using the gmx_MMPBSA tool to quantify the contribution of individual amino acid residues to ligand binding. In this analysis, per-residue energy decomposition included individual energy terms and the total binding free energy contribution. The selection of key residues was based on the total MMPBSA binding free energy contribution rather than any single energy component. Specifically, residues were ranked by their total contribution values, and the three residues with the most negative total binding free energy contributions were identified as key residues (Fig. 14A). Furthermore, we monitored the distances between the ligand and the side chains of these key residues throughout the 100 ns molecular dynamics simulations to evaluate their dynamic stability. Results indicate that across all simulations (using different random initial velocities), the distance between the ligand and active site residues remained stable below 0.5 nm throughout the simulation period with minimal fluctuations (Fig. 14B, Fig. S7). Specifically, in the third simulation, the distance between the ligand and residues 139GLU and 253SER in the AURKA-BaP complex slowed upward trend during the 0–50 ns interval, although it was sustained below 2.5. Following a brief fluctuation near 50 ns, this distance rapidly recovered to within 2.5 nm and was maintained in a stable state for the remainder of the simulation (Fig. S8). These results consistently demonstrate that BaP forms stable and reliable interaction complexes with the four target proteins.

## Discussion

Exposure to exogenous carcinogens has substantially risen sharply with environmental pollution and unhealthy lifestyle patterns, and tumorigenesis is thought to be promoted through two principal mechanisms: (i) induction of DNA damage and disruption of genomic stability (Jeffy, Chirnomas & Romagnolo, 2002; Sadikovic & Rodenhiser, 2006), and (ii) alteration of the tumor immune microenvironment (Sweeney, Lazennec & Vogel, 2022). As a typical environmental carcinogen and representative polycyclic aromatic hydrocarbon (PAH), exposure levels of BaP are positively correlated with BC incidence risk (Desnavailles et al., 2024). Reports indicate that BaP from sources such as cigarette smoke can be metabolized into diol epoxides with steric hindrance, which are potent breast carcinogens (Hecht, 2002). BaP is thought to exert its carcinogenic effects primarily through an aryl hydrocarbon receptor (AhR)–dependent dual mechanism: (i) genotoxicity following metabolic activation, and (ii) suppression of BRCA1 transcription, promoting cell proliferation and inhibiting apoptosis (Hockings et al., 2006; Kemp et al., 2006). The dual properties of BaP—genotoxicity and estrogenic activity—have been the focus of considerable attention in mechanistic studies of BC. However, the molecular mechanisms linking environmental BaP exposure to BC occurrence have yet to be systematically elucidated.

Our study employed a network toxicology-based framework, integrating ChEMBL, PharmMapper, SEA, and GEO databases, and identified 216 candidate targets associated with BaP exposure and BC. Enrichment analyses revealed significant involvement of the MAPK signaling cascade, which governs multiple malignant phenotypes, including cell proliferation, adhesion, differentiation, and apoptosis (Guo et al., 2020). Abnormal activation of this cascade has been reported to promote BC cell proliferation through ERK1/2 and JNK1/2 activation, thereby regulating downstream transcription factors (Naeem et al., 2022). Wu et al. (2022) reported that, in triple-negative breast cancer (TNBC), fibroblast growth factor receptor (FGFR) inhibitors significantly reduced proliferation and migration in cancer-associated fibroblasts by inhibiting the MAPK/ERK pathway. This inhibition disrupted the physicochemical barriers within the tumor immune microenvironment, thereby facilitating T-cell infiltration. Collectively, these findings suggest that BaP may promote malignant transformation and progression of BC through activation of the MAPK signaling pathway.

Using multiple machine-learning algorithms, seven core targets were further identified, and their associations with BC were investigated in depth. Compared with adjacent normal tissue, four core targets—KIF11, AURKA, INHBA, and NEK2—showed significantly higher expression in BC tissue. ROC analyses demonstrated robust diagnostic performance (AUC > 0.90) for each of these genes, highlighting their clinical potential as novel BC biomarkers. More importantly, these findings were validated not only in the GSE86374 cohort but also in the TCGA-BRCA dataset, where the ROC-AUC values exceeded 0.9, further underscoring their reliability as biomarkers. However, the observed overexpression and strong diagnostic performance of these genes primarily reflect general mechanisms of BC pathogenesis. The current results do not, by themselves, establish specificity to BaP exposure. Additional studies directly addressing BaP-dependent regulation or effects would be required to determine whether these genes represent BaP-specific targets.

A lncRNA–miRNA–mRNA–pathway regulatory network was constructed using in silico target-prediction databases only; therefore, it should be interpreted as a hypothesis-generating framework rather than confirmatory. Previous studies have demonstrated that the lncRNA-miRNA-mRNA competitive endogenous RNA (ceRNA) network plays a pivotal role in tumorigenesis and cancer progression. For example, LINC00662 upregulates SOX2 expression by competitively binding to miR-144-3p, thereby regulating BC cellular processes (An et al., 2022). Because we did not perform expression-based correlation validation, future experimental validation will be necessary to confirm the proposed regulatory relationships.

GSVA analyses indicated that these genes were primarily enriched in pathways including cell-cycle regulation, DNA replication, CAMs, oxidative phosphorylation, and cytokine–cytokine receptor interactions. Notably, dysregulation of the cell-cycle is a hallmark of BC and targeting cell-cycle regulators (*e.g.*, CDK2/4/7) has been shown to overcome drug resistance and enhance immunotherapy efficacy (Fuentes-Antras, Bedard & Cescon, 2024). Immune infiltration analysis further demonstrated that these genes were positively correlated with CD4^+^ memory T cells, Tfh cells, macrophage subsets (M0/M1/M2), and mast cells, but were negatively correlated with plasma cells, activated CD8^+^ T cells, and NK cells. These factors have been widely recognized as risk factors in BC research. It has been demonstrated that tumor-associated macrophages (TAMs) consist of pro-inflammatory, antitumor M1 and pro-tumorigenic M2 subsets, which, through distinct mechanisms, contribute to the progression and metastasis of BC (Munir et al., 2021; Li et al., 2022). Overall, our analyses imply potential associations between these genes and immune cell infiltration patterns in the BC tumor microenvironment, further experimental validation is needed to establish whether these correlations influence tumorigenesis and immune evasion processes.

Molecular docking results indicate that BaP can form stable complexes with four key proteins—KIF11, AURKA, INHBA, and NEK2. For KIF11, AURKA, and INHBA, BaP showed more favorable predicted docking scores than the corresponding positive-control inhibitors, suggesting relatively stronger predicted binding tendencies in silico and supporting these targets as candidates for further investigation. Importantly, we note that Vina docking scores are approximate scoring functions primarily intended for relative ranking rather than quantitative estimates of binding affinity; therefore, these results should be interpreted as hypothesis-generating and require experimental confirmation. These proteins are well-established contributors to BC pathogenesis, where their aberrant expression or activation drives key oncogenic processes. For instance, KIF11, a member of the kinesin family, has been shown to disrupt mitosis, leading to genomic instability and thereby promoting carcinogenesis (Asbaghi et al., 2017). KIF11 has also been shown to enhance breast cancer stem-cell properties *via* Wnt/β-catenin activation, which is linked to poor prognosis in BC (Pei et al., 2019; Zhou et al., 2019). AURKA, a critical oncoprotein involved in BC progression, has been shown to exert multiple oncogenic effects during tumor initiation and development (Jalalirad et al., 2021). AURKA fulfills an essential role in cell-cycle progression that drives the G2/M transition and entry into mitosis, and it is linked to increased BC risk (Kahl et al., 2022). Through nuclear translocation, AURKA has been reported to enhance breast cancer stem cell properties, thereby conferring a distinct malignant phenotype to tumor cells (Zheng et al., 2016; Peng et al., 2021). This strategy significantly enhanced naringin-induced ferroptosis, thereby sensitizing TNBC to radiotherapy (Guo et al., 2024). INHBA induces epithelial-mesenchymal transition (EMT) by activating the TGF-β signaling pathway, thereby enhancing the invasiveness of breast cancer cells. This functional characteristic makes it a potential diagnostic biomarker (Yu et al., 2021). Silencing of NEK2 has been shown to curb the proliferation, migration, and invasion of BC cells, while perturbations of the ERK/MAPK cascade concomitantly trigger apoptosis (Xing et al., 2021).

BaP is a potent activator of the aryl hydrocarbon receptor (AhR) signaling pathway, and substantial evidence indicates that AhR activation can broadly regulate cell-cycle progression (Hockley et al., 2007; Hamouchene et al., 2011; Ferguson et al., 2024). The core genes identified in this study—AURKA, NEK2, KIF11, and INHBA—are key regulators of these biological processes, providing a mechanistic basis for a potential link between BaP exposure and the dysregulation of these genes in BC. However, direct evidence specifically associating these genes with BaP exposure in the context of BC remains limited. For example, epidemiological studies have linked airborne BaP exposure to increased BC risk, particularly in certain subtypes (*e.g.*, ER+/PR+), though without implicating these specific targets (Amadou et al., 2021). In other cancers, such as non-small cell lung cancer, KIF11 has been identified as a key gene in BPDE (a BaP metabolite)-induced tumorigenesis, suggesting a role in environmental carcinogen-driven malignancy (Ling et al., 2022). AURKA has similarly been implicated in BaP-induced lung carcinogenesis (Zhao, Zhao & Du, 2025). Coupled with our MD simulations showing stable binding features between BaP and the four corresponding proteins, these findings support the hypothesis that BaP may act through AhR pathway activation to modulate the expression and/or activity of these core targets, thereby disrupting their normal roles in cell-cycle control and promoting malignant progression. Nevertheless, this hypothesis remains to be validated by subsequent *in vitro* and *in vivo* studies to confirm mechanistic causality.

In summary, key molecular targets associated with BaP exposure and BC development were systematically identified, and its multifaceted mechanisms in driving malignant progression were revealed, including the activation of the MAPK signaling pathway, regulation of the ceRNA network, and remodeling of the tumor immune microenvironment. Importantly, stable interactions between BaP and proteins such as KIF11, AURKA, INHBA, and NEK2 were confirmed, offering a structural basis for understanding their functional roles. Collectively, this work enhances current knowledge of BaP’s carcinogenic potential and suggests new directions for biomarker discovery and intervention strategies.

Nevertheless, several limitations must be acknowledged. First, this study was primarily based on integrative bioinformatic analyses of publicly available transcriptomic datasets and in silico modeling. Therefore, the functional roles of the candidate hub genes and the mechanistic involvement of the implicated pathways (*e.g.*, MAPK and RAS signaling) were not directly validated *in vitro* or *in vivo*. Second, key exposure characteristics, such as the dose–response relationship, exposure duration, and interindividual variability in BaP metabolism and susceptibility, were not incorporated into the current analytical framework. Future research should aim to incorporate these exposure characteristics and quantitatively assess their effects on gene expression and carcinogenesis. Third, the immune microenvironment assessment was inferred from publicly available bulk-transcriptomic datasets using CIBERSORT-based deconvolution. As such, the observed associations between BaP-related signatures and immune-cell infiltration patterns should be interpreted as correlative rather than causal, and they require validation in independent cohorts and experimental systems with direct immune profiling. Finally, although molecular docking, binding free-energy estimation, and molecular dynamics simulations suggested a potential for BaP to interact with the selected target proteins, these in silico results alone do not provide sufficient evidence to claim a direct biological effect. Despite these limitations, we significantly strengthened the reliability and clinical relevance of the core gene findings by performing external validation using the large, independent TCGA-BRCA clinical dataset. This step partially compensates for the limitations of purely computational research and provides strong data support for subsequent targeted experimental validation.

Future investigations should aim to experimentally validate the identified targets and pathways in biological models. Coupled with a large-scale prospective cohort to establish causal relationships between BaP exposure and BC risk. Furthermore, multi-omics integration—including epigenomic, transcriptomic, and proteomic data—will be critical to elucidate the complex regulatory networks underlying BaP-induced carcinogenesis. Such approaches are expected to clarify dynamic immune mechanisms and reinforce the biological plausibility of BaP’s carcinogenic effects. Ultimately, this work establishes the foundation for creating precise prevention and control strategies against environmental carcinogens.

##  Supplemental Information

10.7717/peerj.21346/supp-1 Supplemental Information 1 Grid box definition and AutoDock Vina parameters used for molecular docking

10.7717/peerj.21346/supp-2 Supplemental Information 2 The intersection gene of BaP and BC

10.7717/peerj.21346/supp-3 Supplemental Information 3 The results of enrichment analysis

10.7717/peerj.21346/supp-4 Supplemental Information 4 Differentially expressed genes identified in breast cancer

10.7717/peerj.21346/supp-5 Supplemental Information 5 Utilizing LASSO analysis and SVM-RFE algorithm to identify feature genes

10.7717/peerj.21346/supp-6 Supplemental Information 6 Expression comparisons between tumor and adjacent normal tissues in TCGA-BRCA

10.7717/peerj.21346/supp-7 Supplemental Information 7 Binding energies and RMSD values for BaP and inhibitors docked to the four core target proteins

10.7717/peerj.21346/supp-8 Supplemental Information 8 Bioinformatics analysis code

10.7717/peerj.21346/supp-9 Supplemental Information 9 Workflow and study design overview

10.7717/peerj.21346/supp-10 Supplemental Information 10 Differential expression of key BaP-related targets in breast cancer The DEG dataset was analyzed for seven candidate targets: (A) AURKA, (B) EGFR, (C) INHBA, (D) KIF11, (E) NEK2, (F) NR3C2, and (G) YBX3.

10.7717/peerj.21346/supp-11 Supplemental Information 11 RMSD and Rg trajectory of BaP-ligand complex of replica 2 (A) AURKA-BaP complex, (B) INHBA-BaP complex, (C) KIF11-BaP complex, and (D) NEK2-BaP complex.

10.7717/peerj.21346/supp-12 Supplemental Information 12 SASA and RMSF trajectory of BaP-ligand complex of replica 2 (A) AURKA-BaP complex, (B) INHBA-BaP complex, (C) KIF11-BaP complex, and (D) NEK2-BaP complex.

10.7717/peerj.21346/supp-13 Supplemental Information 13 RMSD and Rg trajectory of BaP-ligand complex of replica 3 (A) AURKA-BaP complex, (B) INHBA-BaP complex, (C) KIF11-BaP complex, and (D) NEK2-BaP complex.

10.7717/peerj.21346/supp-14 Supplemental Information 14 SASA and RMSF trajectory of BaP-ligand complex of replica 3 (A) AURKA-BaP complex, (B) INHBA-BaP complex, (C) KIF11-BaP complex, and (D) NEK2-BaP complex.

10.7717/peerj.21346/supp-15 Supplemental Information 15 The ligand-active site residue distance of replica 2 (A) AURKA-BaP complex, (B) INHBA-BaP complex, (C) KIF11-BaP complex, and (D) NEK2-BaP complex.

10.7717/peerj.21346/supp-16 Supplemental Information 16 The ligand-active site residue distance of replica 3 (A) AURKA-BaP complex, (B) INHBA-BaP complex, (C) KIF11-BaP complex, and (D) NEK2-BaP complex.

10.7717/peerj.21346/supp-17 Supplemental Information 17 Dynamic simulation raw data (replica 1)

10.7717/peerj.21346/supp-18 Supplemental Information 18 Dynamic simulation raw data (replica 2)

10.7717/peerj.21346/supp-19 Supplemental Information 19 Dynamic simulation raw data (replica 3)

## Additional Information and Declarations

### Data Availability

The following information was supplied regarding data availability: The code, raw data, and the bioinformatics analysis code are available in the Supplemental Files.

## References

[ref-1] Adeniran JA, Abdulraheem MO, Ameen HA, Odediran ET, Yusuf MO (2021). Source identification and health risk assessments of polycyclic aromatic hydrocarbons in settled dusts from different population density areas of Ilorin, Nigeria. Environmental Monitoring and Assessment.

[ref-2] Amadou A, Praud D, Coudon T, Deygas F, Grassot L, Faure E, Couvidat F, Caudeville J, Bessagnet B, Salizzoni P, Gulliver J, Leffondré K, Severi G, Mancini FR, Fervers B (2021). Risk of breast cancer associated with long-term exposure to benzo[a]pyrene (BaP) air pollution: evidence from the French E3N cohort study. Environment International.

[ref-3] An C, Hu Z, Li Y, Zhao P, Liu R, Zhang Q, Zhu P, Li Y, Wang Y (2022). LINC00662 enhances cell progression and stemness in breast cancer by MiR-144-3p/SOX2 axis. Cancer Cell International.

[ref-4] Asbaghi Y, Thompson LL, Lichtensztejn Z, McManus KJ (2017). *KIF11* silencing and inhibition induces chromosome instability that may contribute to cancer. Genes Chromosomes Cancer.

[ref-5] Barangi S, Ghodsi P, Mehrabi A, Mehri S, Hayes AW, Karimi G (2023). Melatonin attenuates cardiopulmonary toxicity induced by benzo(a)pyrene in mice focusing on apoptosis and autophagy pathways. Environmental Science and Pollution Research International.

[ref-6] Bukowska B, Mokra K, Michałowicz J (2022). Benzo[a]pyrene-environmental occurrence, human exposure, and mechanisms of toxicity. International Journal of Molecular Sciences.

[ref-7] Cai Y, Dai F, Ye Y, Qian J (2025). The global burden of breast cancer among women of reproductive age: a comprehensive analysis. Scientific Reports.

[ref-8] Chen A, Wen S, Liu F, Zhang Z, Liu M, Wu Y, He B, Yan M, Kang T, Lam EW, Wang Z, Liu Q (2021). CRISPR/Cas9 screening identifies a kinetochore-microtubule dependent mechanism for Aurora-A inhibitor resistance in breast cancer. Cancer Communications.

[ref-9] Deng J, Wei L, Chen Y, Li X, Zhang H, Wei X, Feng X, Qiu X, Liang B, Zhang J (2025). Identification of benzo(a)pyrene-related toxicological targets and their role in chronic obstructive pulmonary disease pathogenesis: a comprehensive bioinformatics and machine learning approach. BMC Pharmacology and Toxicology.

[ref-10] Desnavailles P, Praud D, Le Provost B, Kobayashi H, Deygas F, Amadou A, Coudon T, Grassot L, Faure E, Couvidat F, Severi G, Mancini FR, Fervers B, Proust-Lima C, Leffondré K (2024). Trajectories of long-term exposure to PCB153 and Benzo[a]pyrene (BaP) air pollution and risk of breast cancer. Environmental Health.

[ref-11] Duan Z, Yu C, Yang W, Wang W, Zhang Q, Ruan Q, Zhang R, Zhao Y, Yan S (2025). Network toxicology and molecular docking reveal the potential link between acrylamide exposure and breast cancer. Scientific Reports.

[ref-12] Ferguson DT, Taka E, Tilghman SL, Womble T, Redmond BV, Gedeon S, Flores-Rozas H, Reed SL, Soliman K, Kanga K, Darling-Reed SF (2024). The anticancer effects of the garlic organosulfide diallyl trisulfide through the attenuation of B[a]P-induced oxidative stress, AhR expression, and DNA damage in human premalignant breast epithelial (MCF-10AT1) cells. International Journal of Molecular Sciences.

[ref-13] Fuentes-Antras J, Bedard PL, Cescon DW (2024). Seize the engine: emerging cell cycle targets in breast cancer. Clinical and Translational Medicine.

[ref-14] Genchi G, Sinicropi MS, Lauria G, Carocci A, Catalano A (2020). The effects of cadmium toxicity. International Journal of Environmental Research and Public Health.

[ref-15] Gray JM, Rasanayagam S, Engel C, Rizzo J (2017). State of the evidence 2017: an update on the connection between breast cancer and the environment. Environmental Health.

[ref-16] Guo YJ, Pan WW, Liu SB, Shen ZF, Xu Y, Hu LL (2020). ERK/MAPK signalling pathway and tumorigenesis. Experimental and Therapeutic Medicine.

[ref-17] Guo Y, Wang H, Wang X, Chen K, Feng L (2024). Enhancing radiotherapy in triple-negative breast cancer with hesperetin-induced ferroptosis *via* AURKA targeting nanocomposites. Journal of Nanobiotechnology.

[ref-18] Hamouchene H, Arlt VM, Giddings I, Phillips DH (2011). Influence of cell cycle on responses of MCF-7 cells to benzo[a]pyrene. Bmc Genomics.

[ref-19] Hartung T, FitzGerald RE, Jennings P, Mirams GR, Peitsch MC, Rostami-Hodjegan A, Shah I, Wilks MF, Sturla SJ (2017). Systems toxicology: real world applications and opportunities. Chemical Research in Toxicology.

[ref-20] He Z, Hu Y, Zhang Y, Xie J, Niu Z, Yang G, Zhang J, Zhao Z, Wei S, Wu H, Hu W (2024). Asiaticoside exerts neuroprotection through targeting NLRP3 inflammasome activation. Phytomedicine.

[ref-21] Hecht SS (2002). Tobacco smoke carcinogens and breast cancer. Environmental and Molecular Mutagenesis.

[ref-22] Hill J, Hodsdon W (2014). In utero exposure and breast cancer development: an epigenetic perspective. Journal of Environmental Pathology, Toxicology and Oncology.

[ref-23] Hockings JK, Thorne PA, Kemp MQ, Morgan SS, Selmin O, Romagnolo DF (2006). The ligand status of the aromatic hydrocarbon receptor modulates transcriptional activation of BRCA-1 promoter by estrogen. Cancer Research.

[ref-24] Hockley SL, Arlt VM, Brewer D, Te PR, Workman P, Giddings I, Phillips DH (2007). AHR- and DNA-damage-mediated gene expression responses induced by benzo(a)pyrene in human cell lines. Chemical Research in Toxicology.

[ref-25] Huang S (2024). Analysis of environmental pollutant Bisphenol F elicited prostate injury targets and underlying mechanisms through network toxicology, molecular docking, and multi-level bioinformatics data integration. Toxicology.

[ref-26] Huang ML, Hung YH, Lee WM, Li RK, Jiang BR (2014). SVM-RFE based feature selection and Taguchi parameters optimization for multiclass SVM classifier. ScientificWorldJournal.

[ref-27] Jalalirad M, Haddad TC, Salisbury JL, Radisky D, Zhang M, Schroeder M, Tuma A, Leof E, Carter JM, Degnim AC, Boughey JC, Sarkaria J, Yu J, Wang L, Liu MC, Zammataro L, Malatino L, Galanis E, Ingle JN, Goetz MP, D’Assoro AB (2021). Aurora-A kinase oncogenic signaling mediates TGF-beta-induced triple-negative breast cancer plasticity and chemoresistance. Oncogene.

[ref-28] Jeffy BD, Chirnomas RB, Romagnolo DF (2002). Epigenetics of breast cancer: polycyclic aromatic hydrocarbons as risk factors. Environmental and Molecular Mutagenesis.

[ref-29] Jung YS, Kim Y, Cho YR (2022). Comparative analysis of network-based approaches and machine learning algorithms for predicting drug-target interactions. Methods.

[ref-30] Kahl I, Mense J, Finke C, Boller AL, Lorber C, Győrffy B, Greve B, Götte M, Espinoza-Sánchez NA (2022). The cell cycle-related genes RHAMM, AURKA, TPX2, PLK1, and PLK4 are associated with the poor prognosis of breast cancer patients. Journal of Cellular Biochemistry.

[ref-31] Kemp MQ, Liu W, Thorne PA, Kane MD, Selmin O, Romagnolo DF (2006). Induction of the transferrin receptor gene by benzo[a]pyrene in breast cancer MCF-7 cells: potential as a biomarker of PAH exposure. Environmental and Molecular Mutagenesis.

[ref-32] Kou YL, Liu YJ, Hsu TC, Wu KY, Chen ST, Chen JY, Lin KY, Wang HH, Cheng YT, Chen CC, Cai BH (2025). SB431542, a selective inhibitor of the TGF-β type I receptor, enhances doxorubicin antitumor activity *via* p63 activation in mutant p53 breast cancer cells. Frontiers in Bioscience.

[ref-33] Latchford LP, Perez LS, Conage-Pough JE, Turk R, Cusimano MA, Vargas VI, Arora S, Shienvold SR, Kulp RR, Belverio HM, White FM, Thévenin AF (2025). Differential substrate specificity of ERK, JNK, and p38 MAP kinases toward connexin 43. The Journal of Biological Chemistry.

[ref-34] Li JX, Liao WZ, Huang ZM, Yin X, Ouyang S, Gu B, Guo XG (2023). Identifying effective diagnostic biomarkers for childhood cerebral malaria in Africa integrating coexpression analysis with machine learning algorithm. European Journal of Medical Research.

[ref-35] Li H, Yang P, Wang J, Zhang J, Ma Q, Jiang Y, Wu Y, Han T, Xiang D (2022). HLF regulates ferroptosis, development and chemoresistance of triple-negative breast cancer by activating tumor cell-macrophage crosstalk. Journal of Hematology & Oncology.

[ref-36] Ling J, Wang Y, Ma L, Zheng Y, Tang H, Meng L, Zhang L (2022). KIF11, a plus end-directed kinesin, as a key gene in benzo(a)pyrene-induced non-small cell lung cancer. Environmental Toxicology and Pharmacology.

[ref-37] Malik DE, David RM, Gooderham NJ (2018). Mechanistic evidence that benzo[a]pyrene promotes an inflammatory microenvironment that drives the metastatic potential of human mammary cells. Archives of Toxicology.

[ref-38] Mordukhovich I, Beyea J, Herring AH, Hatch M, Stellman SD, Teitelbaum SL, Richardson DB, Millikan RC, Engel LS, Shantakumar S, Steck SE, Neugut AI, Rossner PJ, Santella RM, Gammon MD (2016). Vehicular traffic-related polycyclic aromatic hydrocarbon exposure and breast cancer incidence: the Long Island Breast Cancer Study Project (LIBCSP). Environmental Health Perspectives.

[ref-39] Muley VY (2025). Functional insights through gene ontology, disease ontology, and KEGG pathway enrichment. Methods in Molecular Biology.

[ref-40] Munir MT, Kay MK, Kang MH, Rahman MM, Al-Harrasi A, Choudhury M, Moustaid-Moussa N, Hussain F, Rahman SM (2021). Tumor-associated macrophages as multifaceted regulators of breast tumor growth. International Journal of Molecular Sciences.

[ref-41] Myers JN, Harris KL, Rekhadevi PV, Pratap S, Ramesh A (2021). Benzo(a)pyrene-induced cytotoxicity, cell proliferation, DNA damage, and altered gene expression profiles in HT-29 human colon cancer cells. Cell Biology and Toxicology.

[ref-42] Naeem M, Iqbal MO, Khan H, Ahmed MM, Farooq M, Aadil MM, Jamaludin MI, Hazafa A, Tsai WC (2022). A review of twenty years of research on the regulation of signaling pathways by natural products in breast cancer. Molecules.

[ref-43] Pei YY, Li GC, Ran J, Wan XH, Wei FX, Wang L (2019). Kinesin family member 11 enhances the self-renewal ability of breast cancer cells by participating in the Wnt/beta-catenin pathway. Journal of Breast Cancer.

[ref-44] Peng F, Xu J, Cui B, Liang Q, Zeng S, He B, Zou H, Li M, Zhao H, Meng Y, Chen J, Liu B, Lv S, Chu P, An F, Wang Z, Huang J, Zhan Y, Liao Y, Lu J, Xu L, Zhang J, Sun Z, Li Z, Wang F, Lam EW, Liu Q (2021). Oncogenic AURKA-enhanced N(6)-methyladenosine modification increases DROSHA mRNA stability to transactivate STC1 in breast cancer stem-like cells. Cell Research.

[ref-45] R Core Team (2025). https://www.r-project.org.

[ref-46] Sadikovic B, Rodenhiser DI (2006). Benzopyrene exposure disrupts DNA methylation and growth dynamics in breast cancer cells. Toxicology and Applied Pharmacology.

[ref-47] Sinelnikova A, Spoel DV (2021). NMR refinement and peptide folding using the GROMACS software. Journal of Biomolecular NMR.

[ref-48] Stading R, Gastelum G, Chu C, Jiang W, Moorthy B (2021). Molecular mechanisms of pulmonary carcinogenesis by polycyclic aromatic hydrocarbons (PAHs): implications for human lung cancer. Seminars in Cancer Biology.

[ref-49] Sun C, Zhu B, Zhu S, Zhang L, Du X, Tan X (2021). Risk factors analysis of bone mineral density based on lasso and quantile regression in America during 2015–2018. International Journal of Environmental Research and Public Health.

[ref-50] Sung H, Ferlay J, Siegel RL, Laversanne M, Soerjomataram I, Jemal A, Bray F (2021). Global cancer statistics 2020: GLOBOCAN estimates of incidence and mortality worldwide for 36 cancers in 185 countries. CA: A Cancer Journal for Clinicians.

[ref-51] Sweeney C, Lazennec G, Vogel C (2022). Environmental exposure and the role of AhR in the tumor microenvironment of breast cancer. Frontiers in Pharmacology.

[ref-52] Thankamony AP, Murali R, Karthikeyan N, Varghese BA, Teo WS, McFarland A, Roden DL, Holliday H, Konrad CV, Cazet A, Dodson E, Yang J, Baker LA, George JT, Levine H, Jolly MK, Swarbrick A, Nair R (2020). Targeting the Id1-Kif11 axis in triple-negative breast cancer using combination therapy. Biomolecules.

[ref-53] Wang H, Xiao S, Tang Y, Han K, Zhang Z, Jin Y, Shen F (2020). Activation of MAPK and Cyclin D1/CDK4 in malignant transformation of human embryonic lung fibroblasts induced by silica and benzopyrene. Asian Pacific Journal of Cancer Prevention.

[ref-54] Wang Z, Wang Y (2025). Mechanism exploration of di(2-ethylhexyl) phthalate (DEHP)-induced breast cancer *via* network toxicology and molecular docking analysis. Scientific Reports.

[ref-55] Wenjie W, Rui L, Pengpeng Z, Chao D, Donglin Z (2025). Integrated network toxicology, machine learning and molecular docking reveal the mechanism of benzopyrene-induced periodontitis. BMC Pharmacology and Toxicology.

[ref-56] White AJ, Bradshaw PT, Hamra GB (2018). Air pollution and breast cancer: a review. Current Epidemiology Reports.

[ref-57] Wu S, Powers S, Zhu W, Hannun YA (2016). Substantial contribution of extrinsic risk factors to cancer development. Nature.

[ref-58] Wu Y, Yi Z, Li J, Wei Y, Feng R, Liu J, Huang J, Chen Y, Wang X, Sun J, Yin X, Li Y, Wan J, Zhang L, Huang J, Du H, Wang X, Li Q, Ren G, Li H (2022). FGFR blockade boosts T cell infiltration into triple-negative breast cancer by regulating cancer-associated fibroblasts. Theranostics.

[ref-59] Xi JB, Fang YF, Frett B, Zhu ML, Zhu T, Kong YN, Guan FJ, Zhao Y, Zhang XW, Li HY, Ma ML, Hu W (2017). Structure-based design and synthesis of imidazo[1, 2-a]pyridine derivatives as novel and potent Nek2 inhibitors with *in vitro* and *in vivo* antitumor activities. European Journal of Medicinal Chemistry.

[ref-60] Xing Z, Zhang M, Wang X, Liu J, Liu G, Feng K, Wang X (2021). Silencing of Nek2 suppresses the proliferation, migration and invasion and induces apoptosis of breast cancer cells by regulating ERK/MAPK signaling. Journal of Molecular Histology.

[ref-61] Yadav S, Koka SS, Jain P, Darwhekar GN, Vinchurkar K (2025). Essential database resources for modern drug discovery. Advances in Pharmacology.

[ref-62] Yi L, Wang W, Sun Z, Chen Y, Xiong Z, Ma L, Ye W, Li X (2025). Deciphering the carcinogenic role of benzo[a]pyrene in glioblastoma: insights from network toxicology, single-cell transcriptomics, and Mendelian randomization. Ecotoxicology and Environmental Safety.

[ref-63] Yu Y, Wang W, Lu W, Chen W, Shang A (2021). Inhibin β-A (INHBA) induces epithelial-mesenchymal transition and accelerates the motility of breast cancer cells by activating the TGF-β signaling pathway. Bioengineered.

[ref-64] Zhao Q, Zhao Z, Du K (2025). Exploring the association between air pollutants and non-small cell lung cancer using network toxicology and machine learning. Discover Oncology.

[ref-65] Zheng F, Yue C, Li G, He B, Cheng W, Wang X, Yan M, Long Z, Qiu W, Yuan Z, Xu J, Liu B, Shi Q, Lam EW, Hung MC, Liu Q (2016). Nuclear AURKA acquires kinase-independent transactivating function to enhance breast cancer stem cell phenotype. Nature Communications.

[ref-66] Zhou J, Chen WR, Yang LC, Wang J, Sun JY, Zhang WW, He ZY, Wu SG (2019). KIF11 functions as an oncogene and is associated with poor outcomes from breast cancer. Cancer Research and Treatment.

