# Supplementary figures and images for: Integrative bioinformatics informed by network toxicology and machine learning elucidates the carcinogenic mechanisms of benzo[a]pyrene-induced breast cancer
Source: PeerJ. 2026 Jun 16;14:e21346. doi: 10.7717/peerj.21346 (PMC13281750; doi:10.7717/peerj.21346)

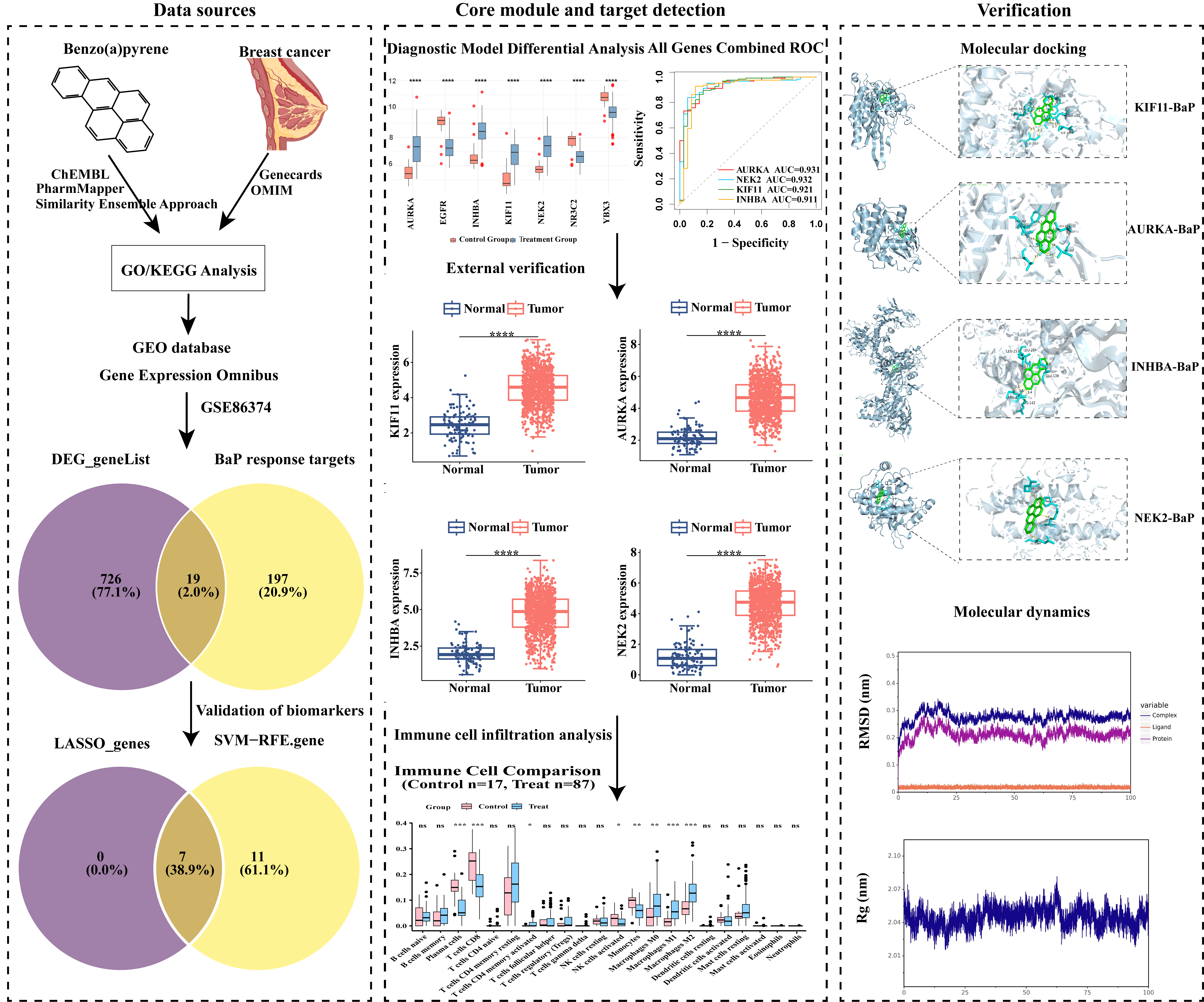

Supplement: Supplemental Information 9 [file peerj-14-21346-s009.png]

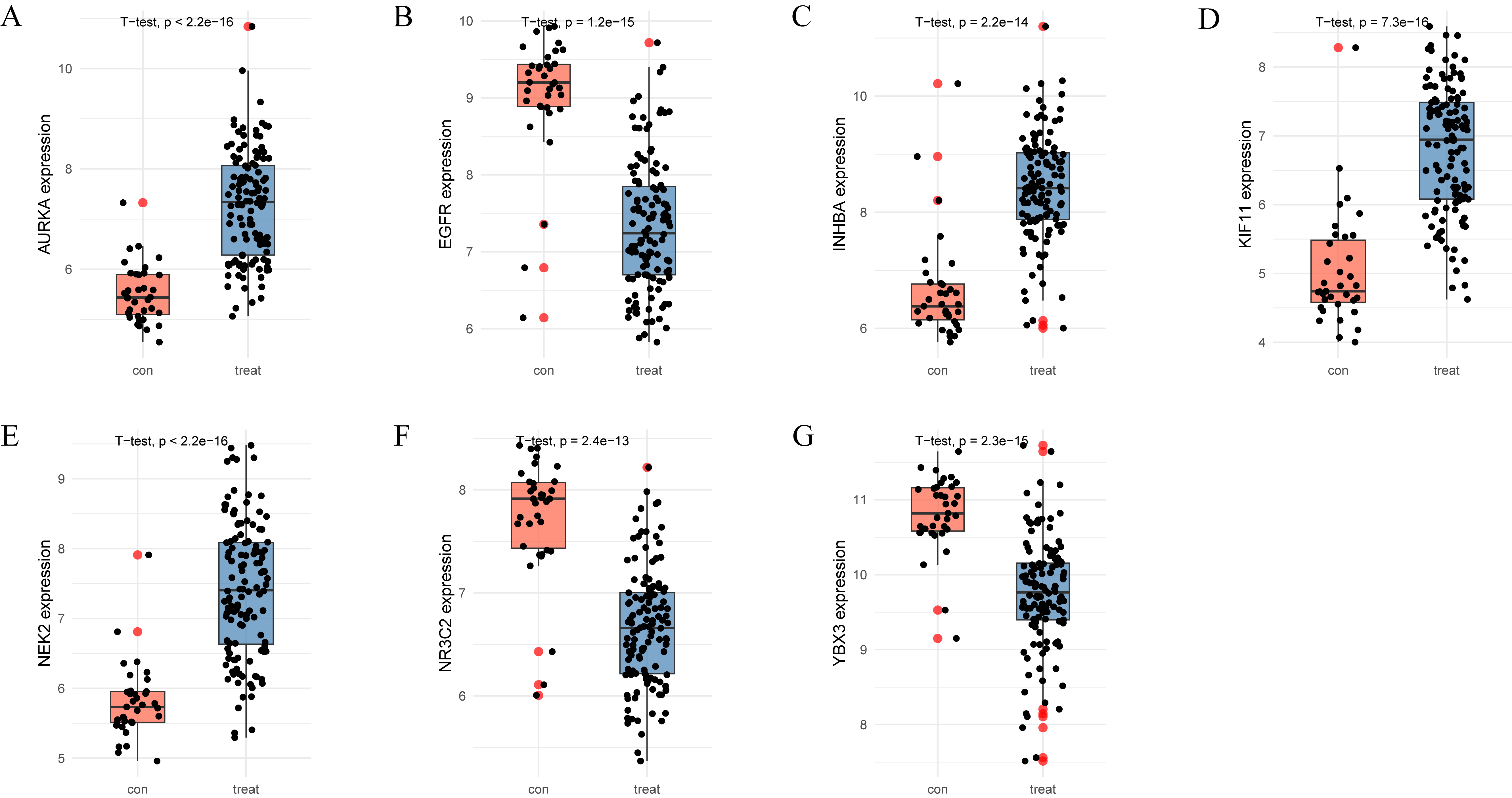

Supplement: Supplemental Information 10 — The DEG dataset was analyzed for seven candidate targets: (A) AURKA, (B) EGFR, (C) INHBA, (D) KIF11, (E) NEK2, (F) NR3C2, and (G) YBX3. [file peerj-14-21346-s010.png]

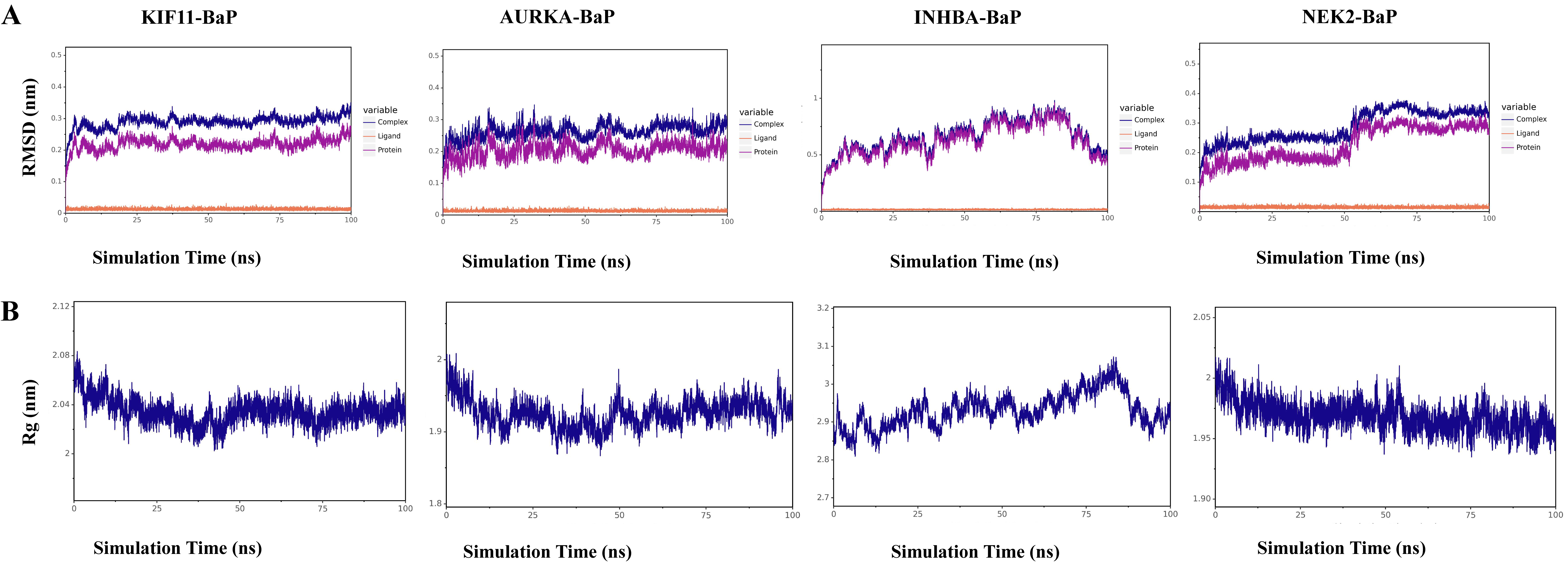

Supplement: Supplemental Information 11 — (A) AURKA-BaP complex, (B) INHBA-BaP complex, (C) KIF11-BaP complex, and (D) NEK2-BaP complex. [file peerj-14-21346-s011.png]

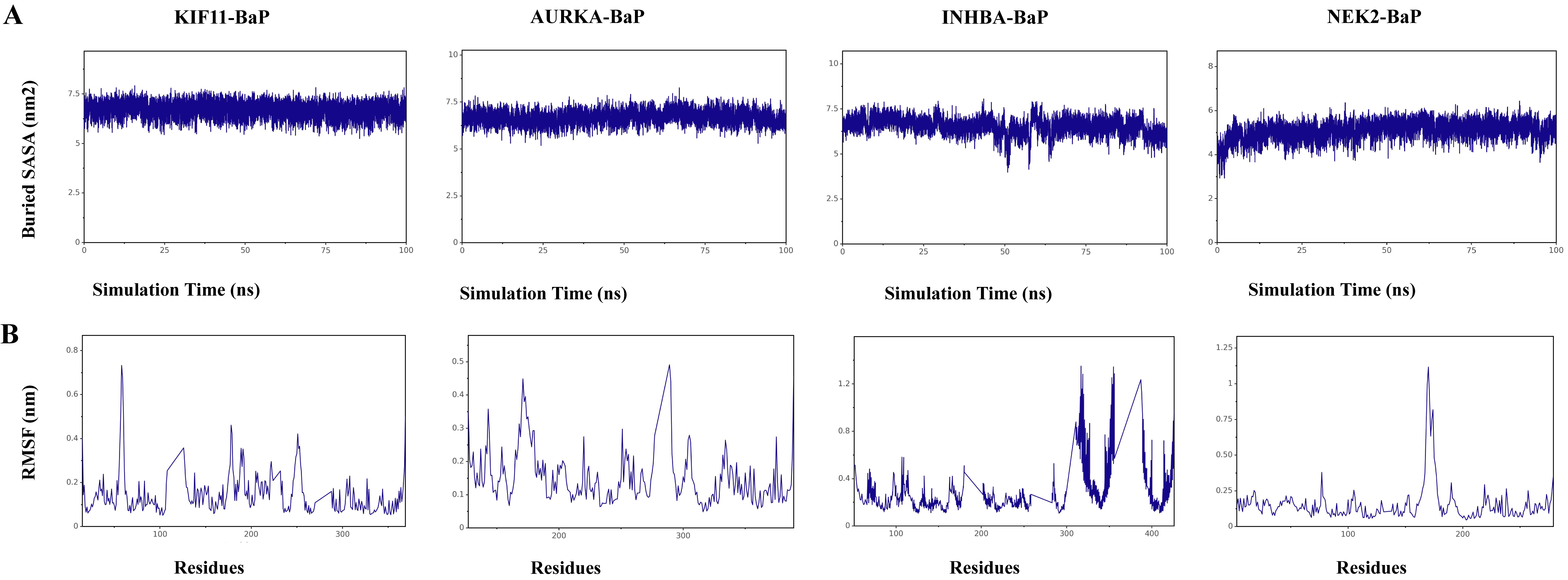

Supplement: Supplemental Information 12 — (A) AURKA-BaP complex, (B) INHBA-BaP complex, (C) KIF11-BaP complex, and (D) NEK2-BaP complex. [file peerj-14-21346-s012.png]

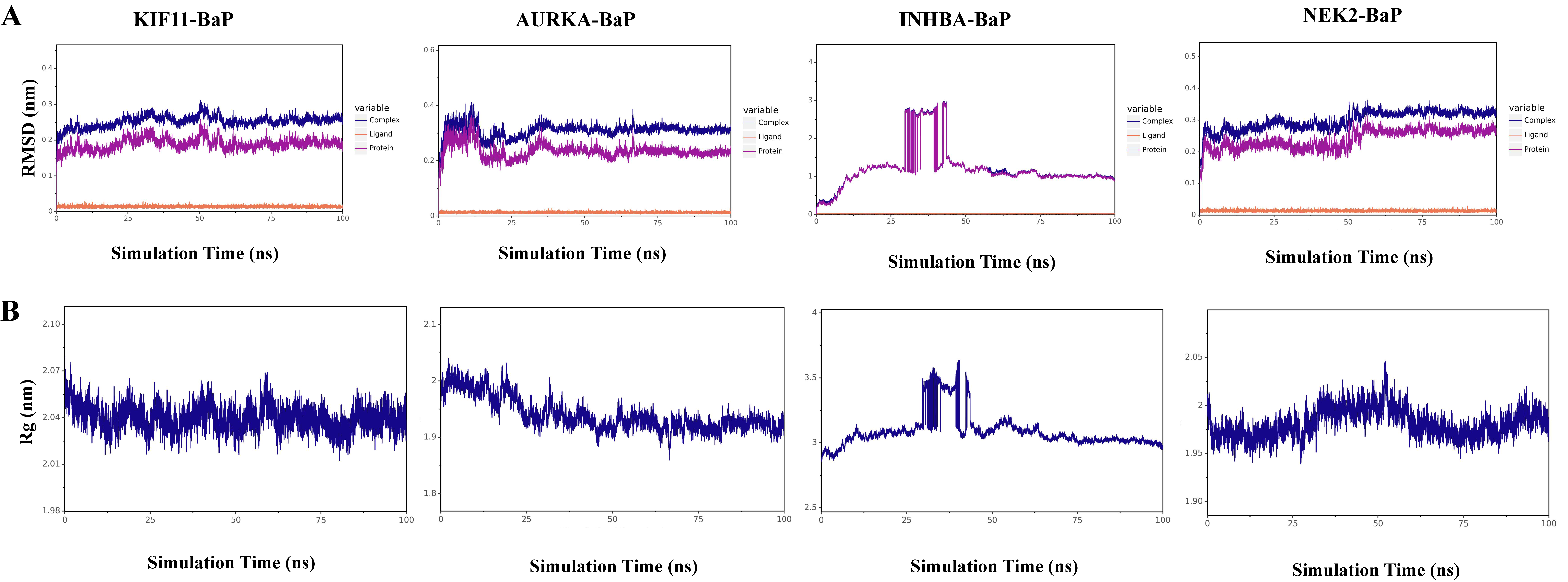

Supplement: Supplemental Information 13 — (A) AURKA-BaP complex, (B) INHBA-BaP complex, (C) KIF11-BaP complex, and (D) NEK2-BaP complex. [file peerj-14-21346-s013.png]

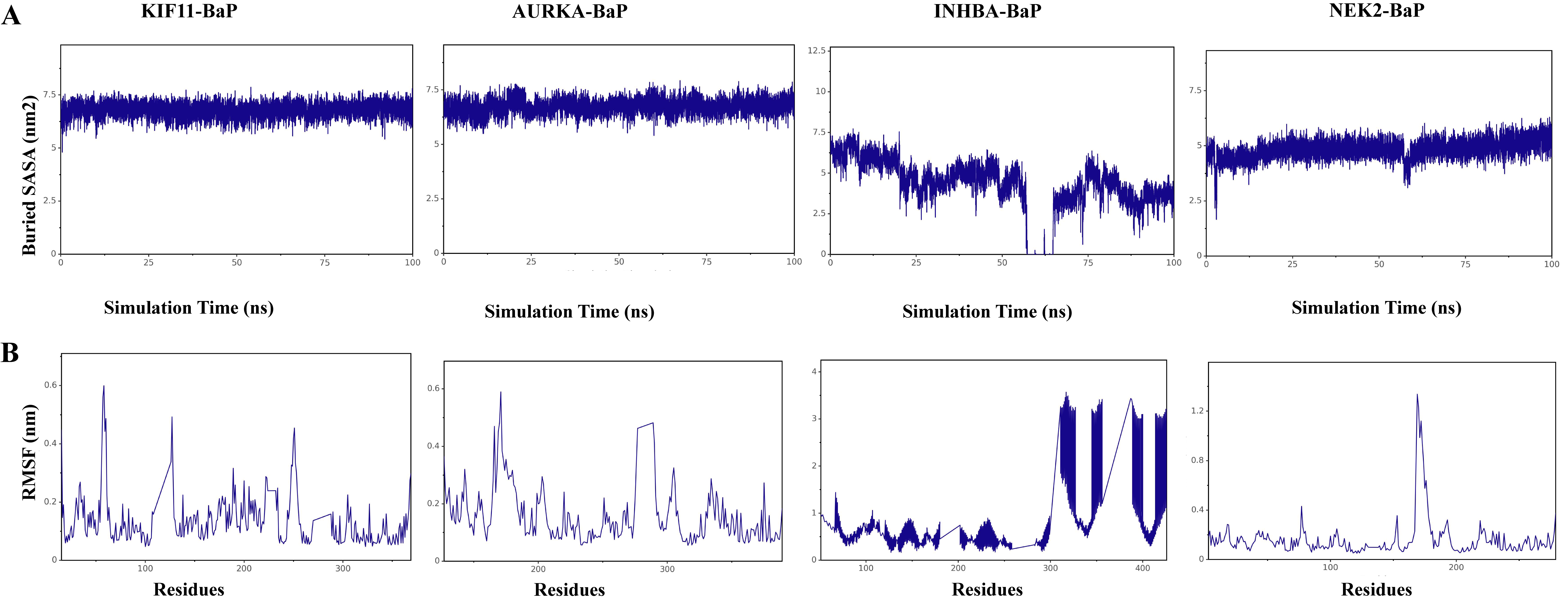

Supplement: Supplemental Information 14 — (A) AURKA-BaP complex, (B) INHBA-BaP complex, (C) KIF11-BaP complex, and (D) NEK2-BaP complex. [file peerj-14-21346-s014.png]

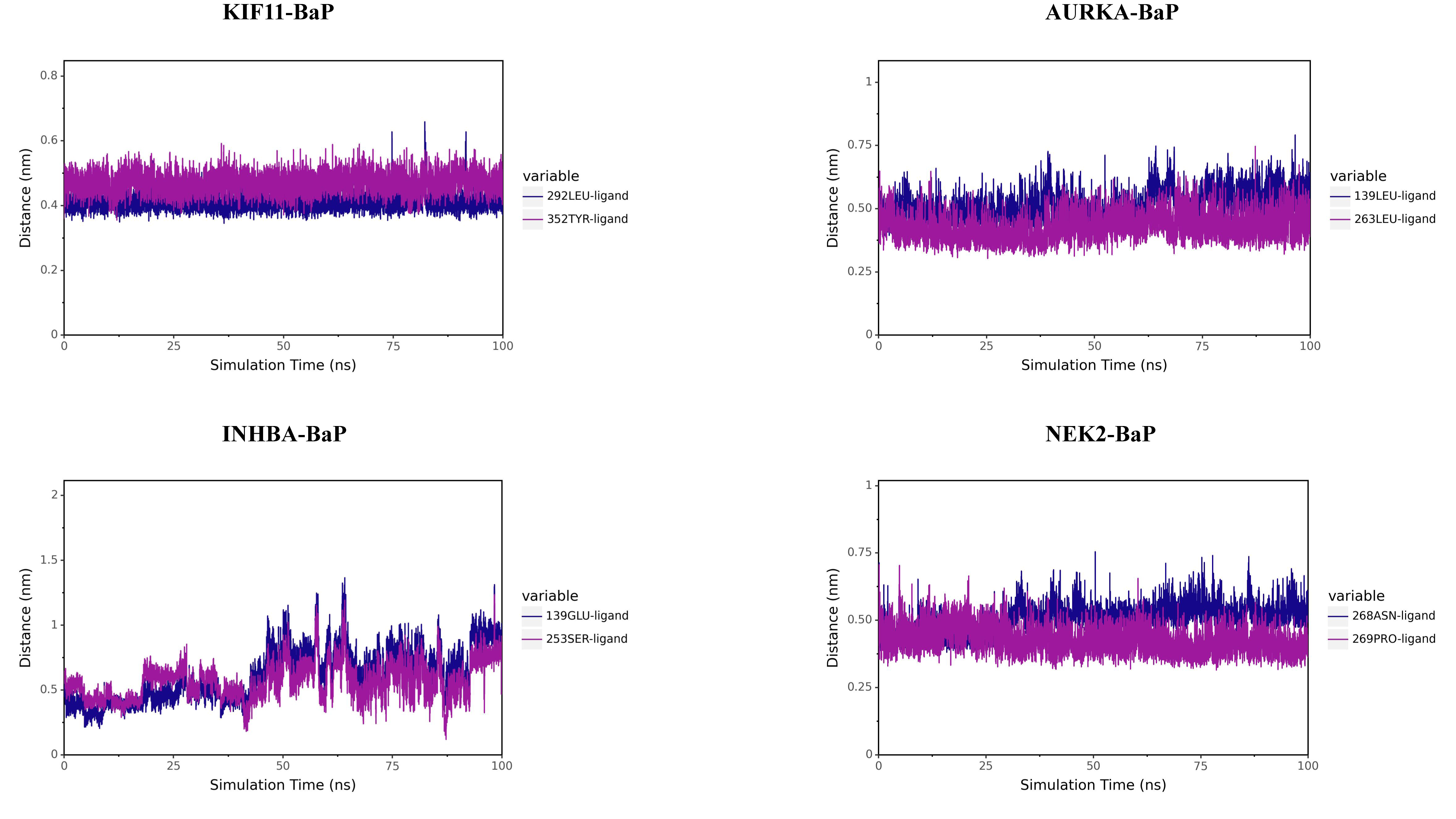

Supplement: Supplemental Information 15 — (A) AURKA-BaP complex, (B) INHBA-BaP complex, (C) KIF11-BaP complex, and (D) NEK2-BaP complex. [file peerj-14-21346-s015.png]

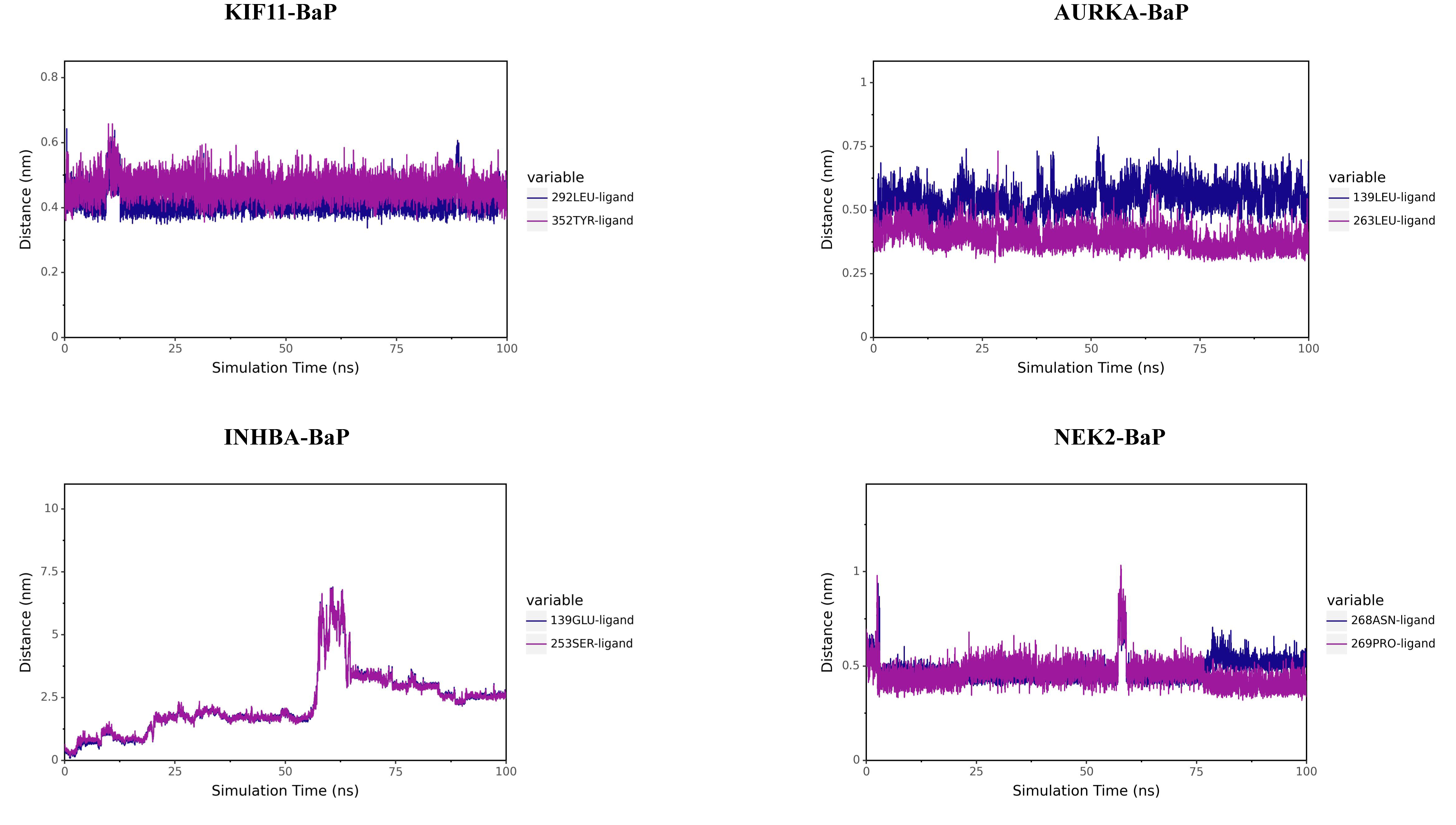

Supplement: Supplemental Information 16 — (A) AURKA-BaP complex, (B) INHBA-BaP complex, (C) KIF11-BaP complex, and (D) NEK2-BaP complex. [file peerj-14-21346-s016.png]
